# BETA-AMYLASE9 is a plastidial nonenzymatic regulator of leaf starch degradation

**DOI:** 10.1093/plphys/kiab468

**Published:** 2021-10-18

**Authors:** Laure C David, Sang-Kyu Lee, Eduard Bruderer, Melanie R Abt, Michaela Fischer-Stettler, Marie-Aude Tschopp, Erik M Solhaug, Katarzyna Sanchez, Samuel C Zeeman

**Affiliations:** Institute of Molecular Plant Biology, Department of Biology, ETH Zurich, Zurich CH-8092, Switzerland

## Abstract

β-Amylases (BAMs) are key enzymes of transitory starch degradation in chloroplasts, a process that buffers the availability of photosynthetically fixed carbon over the diel cycle to maintain energy levels and plant growth at night. However, during vascular plant evolution, the *BAM* gene family diversified, giving rise to isoforms with different compartmentation and biological activities. Here, we characterized BETA-AMYLASE 9 (BAM9) of Arabidopsis (*Arabidopsis thaliana*). Among the BAMs, BAM9 is most closely related to BAM4 but is more widely conserved in plants. BAM9 and BAM4 share features including their plastidial localization and lack of measurable α-1,4-glucan hydrolyzing capacity. BAM4 is a regulator of starch degradation, and *bam4* mutants display a starch-excess phenotype. Although *bam9* single mutants resemble the wild-type (WT), genetic experiments reveal that the loss of BAM9 markedly enhances the starch-excess phenotypes of mutants already impaired in starch degradation. Thus, BAM9 also regulates starch breakdown, but in a different way. Interestingly, *BAM9* gene expression is responsive to several environmental changes, while that of *BAM4* is not. Furthermore, overexpression of BAM9 in the WT reduced leaf starch content, but overexpression in *bam4* failed to complement fully that mutant’s starch-excess phenotype, suggesting that BAM9 and BAM4 are not redundant. We propose that BAM9 activates starch degradation, helping to manage carbohydrate availability in response to fluctuations in environmental conditions. As such, BAM9 represents an interesting gene target to explore in crop species.

## Teaser

A conserved nonenzymatic beta-amylase protein promotes starch breakdown in leaves; mutation of the gene can cause starch accumulation while overexpression can increase nighttime starch use.

## Introduction

Starch is the major storage carbohydrate in plants and is composed of α-1,4- and α-1,6-linked glucose polymers. In leaves, starch is synthetized in chloroplasts during the day and degraded at night, when energy from photosynthesis is unavailable. This pattern of diel turnover is important for optimizing plant growth (Stitt and Zeeman, 2012) and is closely linked with the endogenous circadian clock (Graf et al., 2010; Scialdone et al., 2013; Seki et al., 2017). However, numerous studies analyzing assimilate partitioning and carbohydrate fluxes have illustrated that starch is a dynamic reserve in Arabidopsis (*Arabidopsis thaliana*; Smith and Zeeman, 2020). There are appreciable changes in allocation to, or retrieval from the starch pool during the day (Kölling et al., 2015; Fernandez et al., 2017), in response to environmental fluctuations (Gibon et al., 2004), and in response to various stresses (Kaplan and Guy, 2004; Thalmann et al., 2016; Dong et al., 2018). The regulation of starch turnover to ensure availability of carbohydrates seems to be mediated by changes in both the starch synthesis and degradation rates (Mugford et al., 2014; Thalmann et al., 2016; Fernandez et al., 2017).

The process of starch degradation is complex and required the synergistic actions of several enzymes. Starch forms as semi-crystalline granules, and two dikinases (GLUCAN, WATER DIKINASE and PHOSPHOGLUCAN, WATER DIKINASE) are required to phosphorylate the surface of this structure, thereby disrupting it to facilitate access and activity of glucan hydrolyzing enzymes (Niittylä et al., 2006; Hejazi et al., 2009; Kötting et al., 2009; Santelia et al., 2011). Three classes of glucan hydrolases (α-amylases, BAMs, and debranching enzymes) act in parallel with two phosphoglucan phosphatases (STARCH EXCESS 4 and LIKE STARCH EXCESS FOUR 2) on the starch. Together, these enzymes degrade the starch to maltose and glucose for export to the cytosol while releasing the phosphates introduced by the dikinases. In Arabidopsis, one isoform of α-amylase (AMY3) and two debranching enzymes (ISOAMYLASE 3 and LIMIT DEXTRINASE) are involved in starch degradation (Wattebled et al., 2005; Yu et al., 2005; Delatte et al., 2006; Streb et al., 2012). However, the major degradation product is maltose produced by BAMs. BAMs are exoamylases that hydrolyze the penultimate glucosidic linkage at the nonreducing end of α-1,4-glucan chains to produce β-maltose, hence their name. In Arabidopsis, nine genes encode for BAM-like proteins—more than for any other starch metabolizing enzyme—and other plant genomes contain similar numbers of *BAM* genes (Thalmann et al., 2019). Their characterization in Arabidopsis has so far revealed a surprising degree of sub-functionalization and neo-functionalization (Monroe and Storm, 2018).

Five of the nine proteins (BAM1-3 and BAM5-6) are enzymatically active, four of which are targeted to the plastid (Monroe, 2020). The major isoform in the leaf mesophyll is BAM3, which accounts for 70% of the BAM activity in chloroplasts, followed by BAM1 accounting for 14%, while BAM2 and BAM6 appear to be minor isoforms, whose functions are not yet resolved (Fulton et al., 2008; Monroe et al., 2017; Monroe, 2020). The *bam1*, *bam2*, and *bam6* single mutants have starch contents similar to wild-type (WT) plants, but the loss of BAM3 leads to starch-excess phenotype, which is further exacerbated by the loss of BAM1 (Kaplan and Guy, 2005; Sparla et al., 2006; Fulton et al., 2008; Monroe, 2020). Interestingly, both BAM1 and BAM3 were recently shown to associate with LIKE SEX FOUR1 (LSF1), a nonenzymatic homolog of STARCH EXCESS 4. LSF1 contains a carbohydrate-binding module (CBM) and is proposed to act as a scaffold, helping to localize the BAMs to the starch granule surface (Schreier et al., 2019). The loss of LSF1 also results in a starch-excess phenotype (Comparot-Moss et al., 2010), demonstrating that it plays an important role in starch degradation.

BAM1 is highly expressed in guard cells, where the starch turnover is different from the rest of the leaf, with appreciable starch remaining at the end of the night, when mesophyll cells have nearly exhausted their supplies. At the beginning of the day, BAM1 contributes to the rapid degradation of guard cell starch releasing osmolytes and respiratory substrates needed to bring about stomata opening (Horrer et al., 2016; Flütsch et al., 2020). BAM1 differs from other isoforms, being redox-activated and having an alkaline pH optimum—features consistent with it being active during the daytime in chloroplasts (Sparla et al., 2006; Fulton et al., 2008). BAM1 is also induced in mesophyll cells under osmotic stress conditions (Valerio et al., 2011), promoting starch degradation and supporting the synthesis compatible solutes (Thalmann et al., 2016; Zanella et al., 2016). One active isoform, BAM5, is cytosolic rather than being targeted to the plastid. BAM5 is highly expressed in plants grown in full illumination, when it can contribute the most BAM activity. The protein was reported to be localized in phloem tissues (Wang et al., 1995; Laby et al., 2001), where its hypothesized function is to prevent clogging of sieve tubes by polymerized polysaccharides, although this role has not been confirmed experimentally.

Four of the nine proteins (BAM4 and BAM7-9) appear to have lost enzymatic activity. Two—BAM7 and BAM8—are nonplastidial and are remarkable examples of neo-functionalization. In addition to their BAM2-related BAM domain, these proteins possess a BRASSINAZOLE-RESISTANT1-type DNA binding domain. They are located in the nucleus where they act as transcription factors regulating shoot growth, but do not influence starch metabolism (Reinhold et al., 2011; Soyk et al., 2014). The BAM domain of BAM8 was shown to be required for its transcriptional activation function, leading to the proposition that they act as sugar-sensing domains (Soyk et al., 2014). BAM4 is plastidial and is also an example of neo-functionalization. Compared with active BAMs, BAM4 has numerous substitutions of catalytically important amino acid residues and recombinant BAM4 did not display BAM activity (Fulton et al., 2008). Yet, *bam4* mutants have a starch-excess phenotype and reduced nighttime maltose levels without change in total BAM activity (Fulton et al., 2008; Li et al., 2009). This led to the hypothesis that BAM4 may function as a regulator of starch degradation that influences other enzymes of the starch degradation pathway, although no mechanism has yet been elucidated. Interestingly, the isoform most closely related to BAM4, BAM9, has received relatively little attention. Therefore, we performed a functional analysis of BAM9, which revealed that, like BAM4, BAM9 is a catalytically inactive, chloroplastic protein in Arabidopsis, but that it serves a distinct regulatory function in starch metabolism.

## Results

### BAM9 is a plastidial protein expressed in green tissues

**Figure 1:**
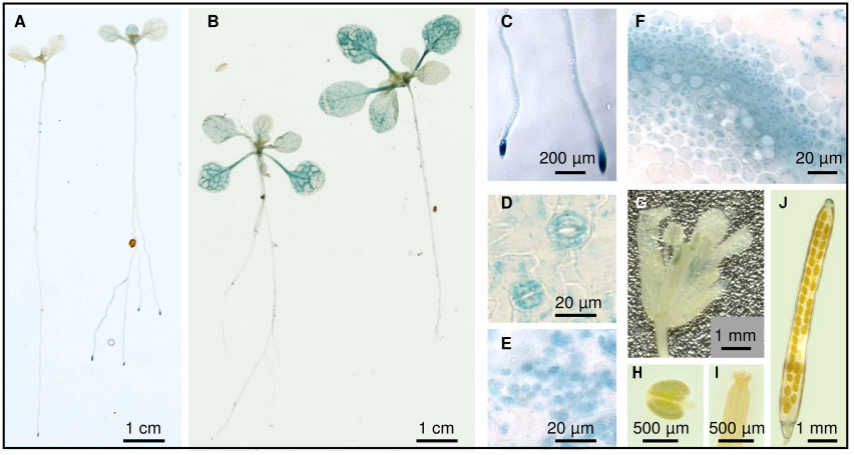
Expression pattern of BAM9 in different organs of Arabidopsis. Genomic DNA containing the BAM9 promoter and the sequence encoding the first 204 amino acids of the BAM9 protein was cloned in frame with the reporter *GUS* gene. Expression patterns in different organs were visualized in the T3 generation. A, seedling stage, (B) mature plants, (C) root tip (enlargement of a plant imaged in (A), (D) stomata of the abaxial side of mature leaves, (E) subcellular localization in mesophyll cells, (F) vascular tissues, (G) flowers, (H) anther, (I) pistil, and (J) silique.

We studied the expression pattern and subcellular localization of BAM9 using a transcriptional fusion construct. The β-glucuronidase (GUS) coding sequence (CDS) was placed after and in frame with the 612 first nucleotides of BAM9 CDS. The resulting construct was driven by the native *BAM9* promoter, which comprised 2.3kb of sequence upstream of the start codon (for primer sequences, see Supplemental Dataset S1). Preliminary screening of all T1 transformants was carried out to identify lines with consistent patterns of expression. Figure  1 shows typical results of GUS staining performed on different organs of plants from one such line, harvested at different developmental stages of the T3 generation. In one-week-old seedlings, GUS staining was visible in the root tip and the leaf vasculature (Figure  1, A and C). Later in development, GUS staining was more homogeneously distributed in the shoots (Figure  1B). In leaves, appreciable GUS staining was also detected in stomatal guard cells (Figure  1D). The presence of the first 204 amino acids of BAM9 protein allowed us to examine via light microscopy the subcellular localization of the fusion protein. GUS staining appeared to be localized to chloroplasts in mesophyll cells, supporting previous predictions that BAM9 is targeted in this organelle (Figure  1E;Stettler, 2009; Monroe and Storm, 2018). Analysis of leaf cross sections revealed that BAM9 was also expressed around the vascular tissues (Figure  1F). In reproductive organs, no GUS staining was detected in the pistil, but it was apparent in the anthers (Figure  1, G–I). After fertilization, during silique development, GUS staining was detected in the silique receptacle (Figure  1J). We also performed quantitative reverse transcription PCR (RT-qPCR) analysis over a 24-h time course, which showed that, in the shoots, *BAM9* expression increases during the night to reach its peak expression near dawn (Supplemental Figure S1), confirming earlier microarray data (Smith et al., 2004).

**Figure 2:**
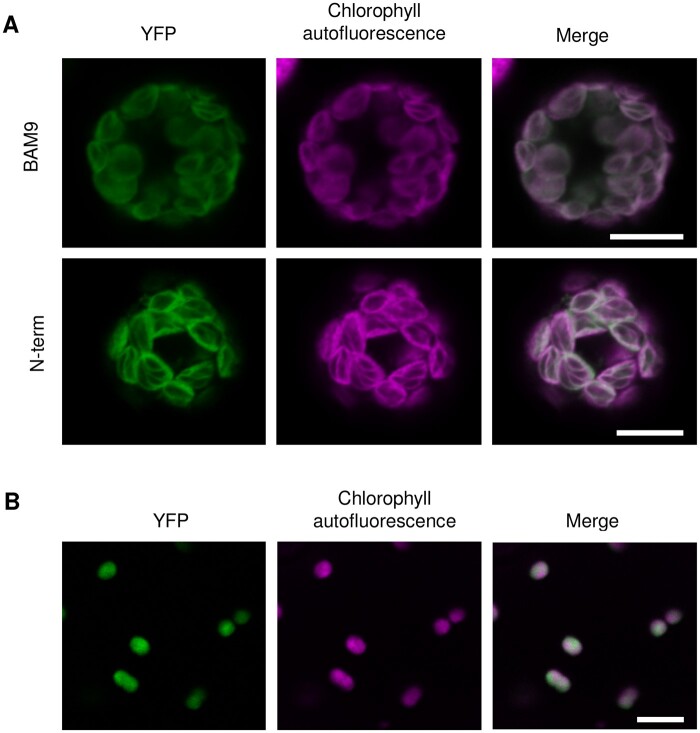
Subcellular Localization of the BAM9 protein. A, Constructs encoding full-length BAM9 protein (upper parts) or its 71-amino acid N-terminal extension (lower parts) fused to YFP at their C-terminal ends were transfected into leaf mesophyll protoplasts from WT Arabidopsis plants. Scale bars, 10 µm. B, Constructs encoding BAM9–YFP protein, as in (A), under control of ubiquitous promoter was stably expressed in WT plants. Scale bars, 10 µm.

We further verified the subcellular localization of BAM9 by producing a construct in which the full-length BAM9 CDS was placed upstream and in-frame with that of yellow fluorescent protein (YFP). This construct, driven by the Arabidopsis Ubiquitin10 promoter, was used both for transient expression studies in leaf mesophyll protoplasts from WT plants and for stable transformation of WT plants. In both cases, YFP fluorescence was exclusively located in chloroplasts (Figure  2), consistent with the GUS staining localization (Figure  1E). Despite our clear localization results, different tools to predict subcellular localization give inconsistent results for BAM9 (SUBA4; Hooper et al., 2017) and algorithms to identify chloroplast transit peptide (ChloroP; Emanuelsson et al., 1999), give negative results. Nevertheless, like the known chloroplastic BAMs (BAM1-3 and BAM6), BAM9 has an N-terminal extension to its family 14 glucosyl hydrolase-like domain of almost 100 amino acids. To assess if this extension serves as a chloroplast transit peptide, we cloned the part of the *BAM9* gene encoding the first 71 amino acids of the protein and inserted it upstream and in-frame with the CDS of YFP. As for the full-length fusion protein, the YFP signal from expression of this construct was detected in chloroplasts (Figure  2A), from which we conclude that BAM9 does have an N-terminal transit peptide.

### BAM9 is catalytically inactive

**Figure 3:**
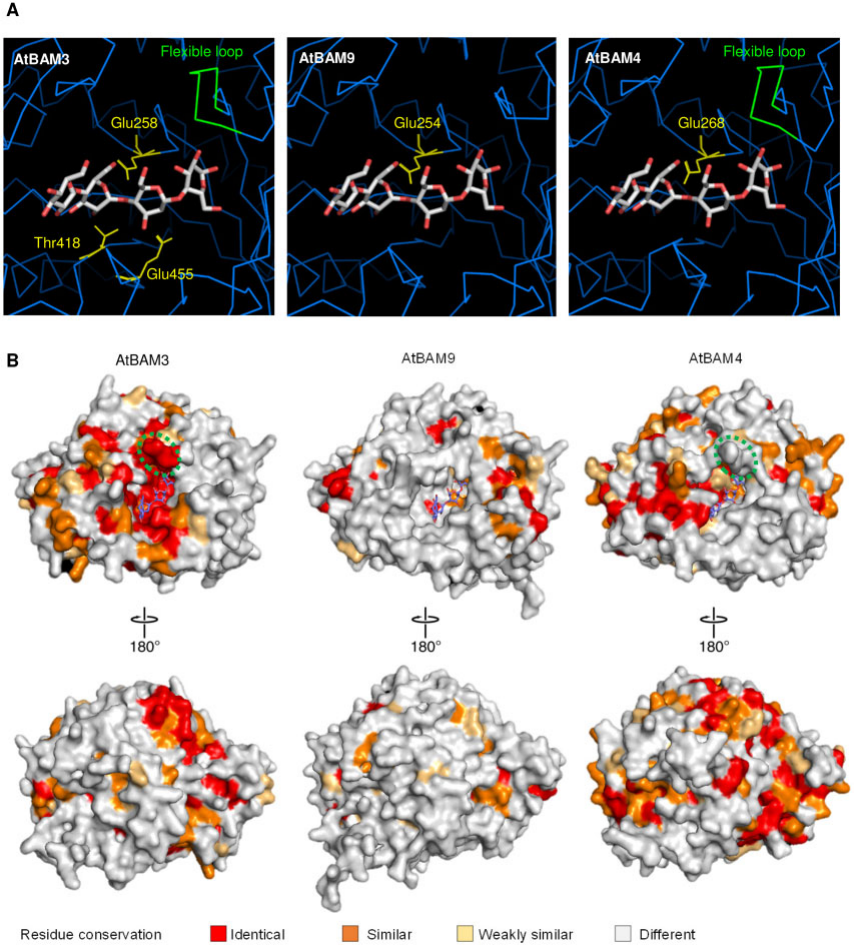
Structural modeling of BAM9 reveals key residues missing in the catalytic domain. A, Detailed view of the predicted BAM catalytic domain of AtBAM3, AtABM4, and AtBAM9 modeled on the crystal structure of the sweet potato BAM bound to maltotetraose (carbons are shown in light gray and oxygens in red) as a template (Vajravijayan et al., 2018). Where present, the flexible loop is highlighted in green, and the three important catalytic residues in yellow. Residue number is based on the position in the sequence of each respective Arabidopsis protein. B, Structure predictions for AtBAM3, AtABM4, and AtBAM9, as in (A) (maltotetraose shown with carbons in dark blue and oxygens in red). Amino acid conservation, determined by aligning orthologous sequences of each Arabidopsis gene (82 sequences for BAM3, 30 sequences for BAM4, and 78 sequences for BAM9) is projected onto the protein surface. If present, the flexible loop is highlighted by a green dotted circle.

Next, we compared the predicted tertiary structures of BAM9, BAM4, and the catalytically active BAM3 proteins. As a template, we used the recently solved crystal structure of sweet potato BAM bound to maltotetraose (Vajravijayan et al., 2018) (accession numbers can be found in Supplemental Dataset S2). Three residues—two glutamic acids and a threonine (Glu187, Glu382, and Thr344 in sweet potato, equivalent to Glu186 and Glu380 and Thr342 in the soybean protein; Kang et al., 2004, 2005) and a flexible loop (amino acids 95–105 and 94–104 in sweet potato and soybean, respectively) have been described to be crucial for hydrolysis. These features are all observed in BAM3 (Figure  3) and the other active Arabidopsis BAMs (BAM1–2, BAM5–6; Fulton et al., 2008). The first of the two glutamic acid residues responsible for catalysis is present in BAM9 and BAM4 but the other is nonconservatively substituted (to a glutamine in BAM9 and an arginine in BAM4). Similarly, the anchoring threonine, which helps mediate substrate binding is nonconservatively substituted in BAM9 and BAM4 (to a proline in both cases). The flexible loop is missing in BAM9, while present but not conserved in sequence in BAM4 (Figure  3A).

Separate multiple sequence alignments were made for each of the modeled BAMs using orthologous sequences encoded in the genomes of other vascular plants (angiosperms and gymnosperms; accession numbers can be found in Supplemental Dataset S3; see also Thalmann et al., 2019). For each modeled protein, we mapped the degree of residue conservation onto the generated surface representation (Figure  3B). This revealed conservation of residues constituting the glucan binding pocket in BAM3 and its orthologs, as expected. However, no such conservation was observed for the comparable region of BAM9 and its orthologs, while the conservation was weak for BAM4 and its orthologs.

**Figure 4:**
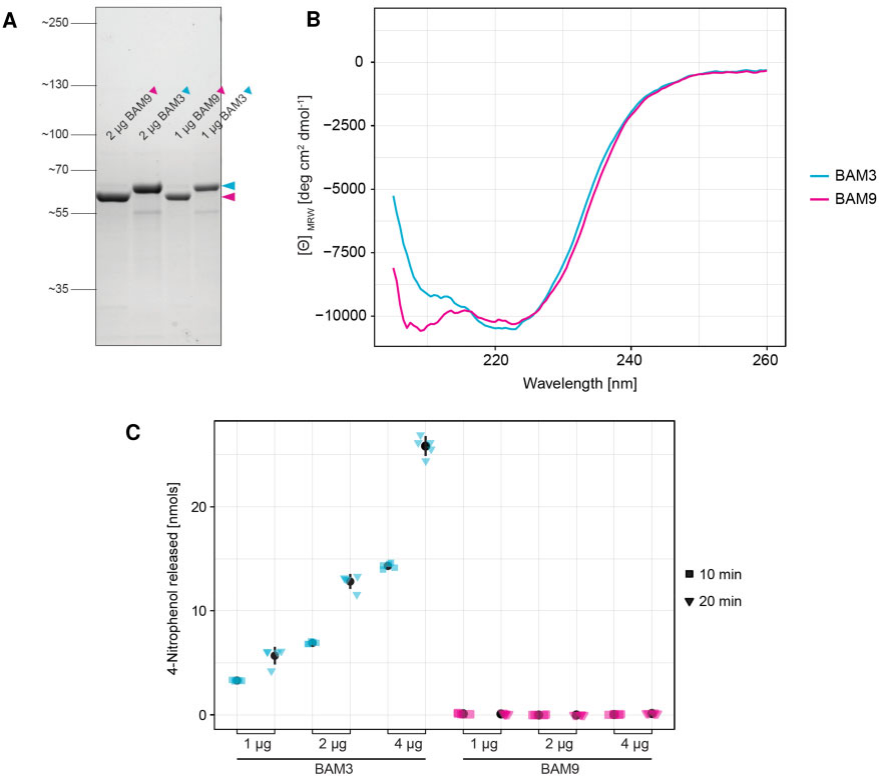
BAM9 recombinant protein is catalytically inactive. A, Purified recombinant proteins analyzed by SDS-polyacrylamide gel electrophoresis and Coomassie staining. Values indicate molecular weight in kilodaltons. B, Far-UV CD spectra of native recombinant BAM3 and BAM9 proteins (both 6×His-tagged, both lacking the N-terminal chloroplast transit peptides), recorded at pH 7.5 and 25°C, showing that both have secondary structure. C, BAM activity assays on BAM9 and BAM3 recombinant proteins using the Betamyl-3 assay reagent at pH 7.5 and 25°C. For each protein, 1, 2, and 4 μg recombinant protein were assayed for either 10 or 20 min, as indicated. Shown is the mean ± sd (*n* = 5 replicate assays) in black. Individual measurements are overlaid in transparent color. All analyses presented were performed on the same protein purification batch, but similar purification and activity data were obtained with independent batches of expressed protein.

Collectively, our bioinformatic and structural modeling results support previous speculations that BAM9 is unlikely to be catalytically active or bind a glucan substrate (Fulton et al., 2008; Thalmann et al., 2019). To test this prediction, we expressed BAM9 (with the first 71 amino acids replaced by a polyhistidine tag) as a recombinant protein in *Escherichia coli*. We also expressed a recombinant tagged version of BAM3 as a control (Figure  4A). After affinity purification, the folding of the proteins was confirmed using circular dichroism (CD) spectroscopy (Figure  4B). Two types of BAM activity assays were then performed. The first assay used the Betamyl-3 reagent which contains *p*-nitrophenyl maltotrioside, a chlorogenic substrate specific for BAM. Recombinant BAM3 protein was active against this substrate (Figure  4B), but no activity was detectable for the recombinant BAM9 protein. In the second assay, the recombinant proteins were incubated together with soluble starch and released glucans were detected with high-performance anion-exchange chromatography with pulsed amperometric detection (HPAEC-PAD). BAM3 released maltose from soluble starch, whereas BAM9 did not (Supplemental Figure S2). No other glucans were detected in the chromatograms. Interestingly, recombinant BAM3 was substantially more active against soluble starch than against the Betamyl-3 reagent (we conservatively estimate 1,000-fold; Supplemental Figure S2).

Next, we investigated whether BAM3 or BAM9 proteins could bind to starch by incubating each recombinant protein with maize starch granules. After several washes, bound protein was eluted with SDS, revealing that a small fraction of the BAM3, but no BAM9 protein remained associated with the granules. This suggests that BAM9 cannot bind strongly to starch (Supplemental Figure S3). Sephadex G10 resin was used as a nonstarch substance to control for nonspecific binding or for protein precipitation, and neither protein was detected after the final wash.

### Loss of BAM9 conditionally alters leaf starch metabolism

**Figure 5:**
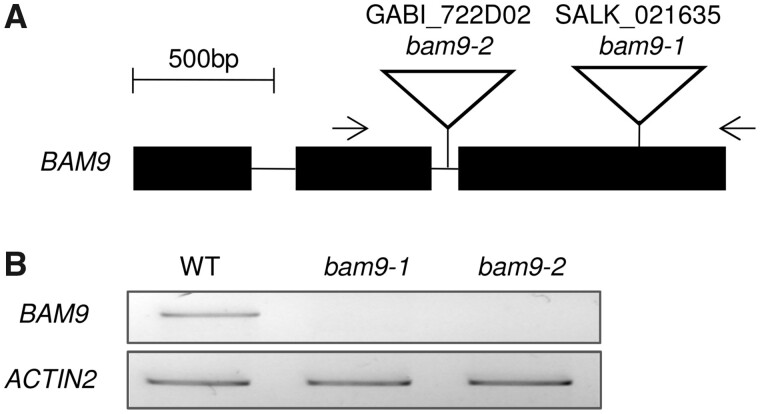
Isolation of *bam9* mutant alleles. A, Structure of *BAM9* gene (exons are shown as filled boxes) and the positions of the T-DNA insertion in *bam9*-1 and *bam9*-2 mutant alleles. B, RT-PCR analysis of *BAM9* gene expression in the *bam9* mutant alleles. *ACTIN2* was used as housekeeping gene.

We investigated the impact of losing BAM9 by isolating and analyzing *bam9* T-DNA insertion mutants (Figure  5A). Two independent alleles were isolated from public mutant collections. Plants homozygous for the insertions were obtained via PCR-based genotyping (for primer sequences, see Supplemental Dataset S1). To confirm the lack of a functional *BAM9* transcript, RT-PCR analysis was performed on extracted mRNA using primers designed to span the insertion site in each case (see Figure  5A;Supplemental Dataset S1). As expected, no PCR product was obtained for each of the *bam9* mutant alleles (Figure  5B). The mutant plants did not differ in appearance from WT plants. Measurements of BAM activity in crude extracts (quantifying maltose released from soluble starch) did not reveal a difference in activity in *bam9*-1 compared to the WT, whereas the *bam3* mutant had a substantial reduction (2.92 ± 0.15, 2.64 ± 0.14, and 0.63 ± 0.07 nmol maltose min^−1^ mg^−1^ fresh weight (FW), respectively).

**Figure 6:**
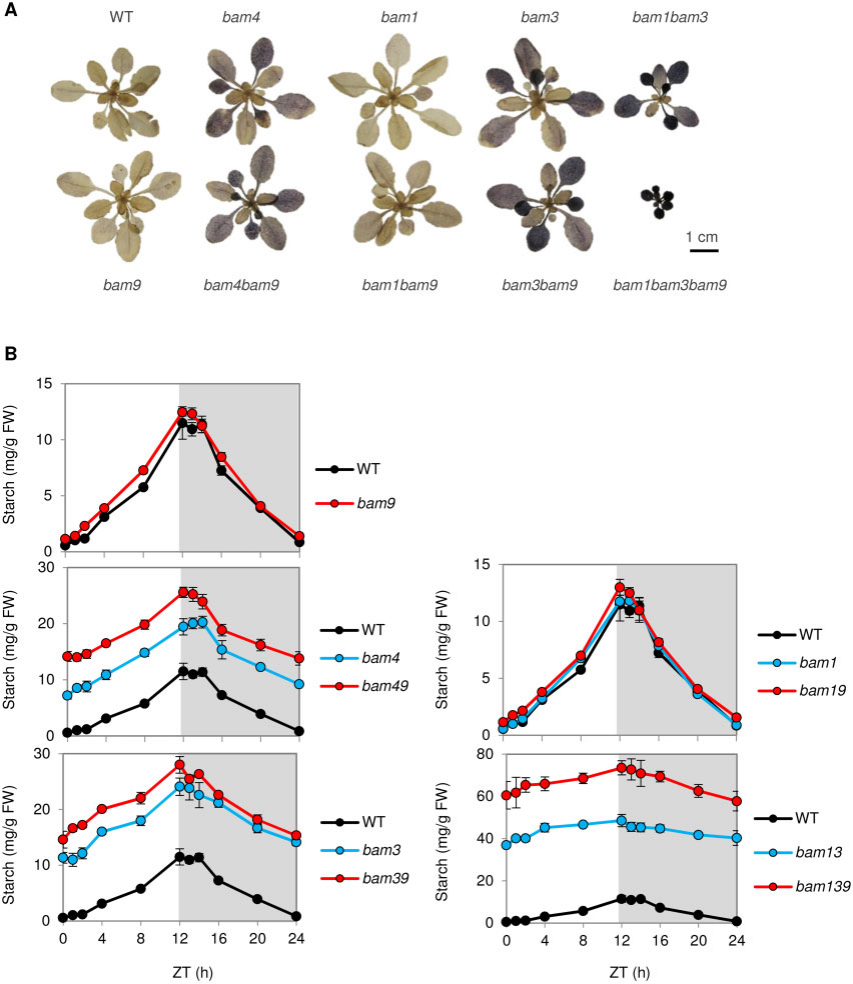
Effect of *bam9*-1 mutation on the starch content in different genetic backgrounds. A, Rosettes of 23-d-old Arabidopsis plants of the indicated genotypes, grown in 12-h d/12-h night regime, were harvested at the end of night, decolorized with 80% (v/v) ethanol, and iodine-stained with Lugol’s solution. All plants were imaged together. Individual rosettes were digitally extracted and positioned for optimal comparison. B, Measurement of starch content in rosettes plants, as in (A) harvested at different time points throughout the diel cycle. *N* = 5 biological replicates ± se. The same data for the WT are shown in each panel (note the differences in the *y*-axis scales). ZT, Zeitgeber time.

We determined whether the mutants were affected in starch turnover in leaves of plants grown in an equinoctial diel cycle. Iodine staining of plants harvested at the end of the night indicated that the *bam9* did not have a starch-excess phenotype, in contrast to previously described *bam4*, *bam3*, and the *bam1bam3* mutant lines (Figure  6A;Fulton et al., 2008). This was confirmed with quantitative measurements over the diel cycle, which also revealed that, overall, the starch content was similar to that of the WT (Figure  6B), although repeated measurements revealed a tendency toward slightly increased starch levels. Given that BAM4 is the closest paralog to BAM9 and that both appear to be enzymatically inactive, we investigated starch content in the double mutant *bam4bam9*. Interestingly, the double mutant exhibited an even more pronounced starch-excess phenotype than the *bam4* single mutant (Figure  6B). Next, we generated additional multiple mutants lacking BAM9 and catalytically active BAM isoforms BAM1 or BAM3. Interestingly, the loss of BAM9 again exacerbated preexisting starch-excess phenotypes. When crossed with *bam1*, which has similar starch content to the WT (Fulton et al., 2008), there was no appreciable difference in the resultant *bam1bam9* double mutant (Figure  6B). In contrast, when crossed with *bam3* the resultant *bam3bam9* double mutant had more starch than *bam3*. Likewise, when crossed with the *bam1bam3* double mutant, the resultant *bam1bam3bam9* triple mutants had much more starch than the already severely affected *bam1bam3* double mutant (Figure  6B). To confirm this phenotypic enhancement effect upon loss of BAM9, we repeated a subset of these crosses using the *bam9*-2 allele. The independently obtained *bam3bam9*, and subsequently the *bam1bam3bam9* mutant combinations yielded similarly enhanced starch-excess phenotypes compared with the parental *bam3* and *bam1bam3* mutants (Supplemental Figure S4).

We observed variation in the absolute starch contents between experiments, which could result from several factors (e.g. slight differences in growth chambers, soil batches, plant management regimes, etc.). However, when we compiled the results of multiple experimental replicates where we measured starch content at the end of the day and at the end of the night, the result of the loss of BAM9 was consistent. The starch-excess phenotype of *bam3*, *bam4*, and *bam1bam3* was enhanced by 56 ± 13%, 80 ± 12%, and 56 ± 14% at the end of the night, and by 27 ± 11%, 22 ± 6%, and 32 ± 11% at the end of the day, respectively, when BAM9 was missing. In each case, the percentage value is the mean ± standard error (SE) of 4–8 experimental replicates, each of which had between 4 and 8 biological replicates. Even in the *bam9* single mutant and in the *bam1bam9* mutant, the end-of-night starch content was consistently higher than in the WT (144 ± 28%) and *bam1* (106 ± 21%), although these differences were typically less than 1 mg g^−1^ FW, and no differences in starch content were apparent at the end of the day.

**Figure 7:**
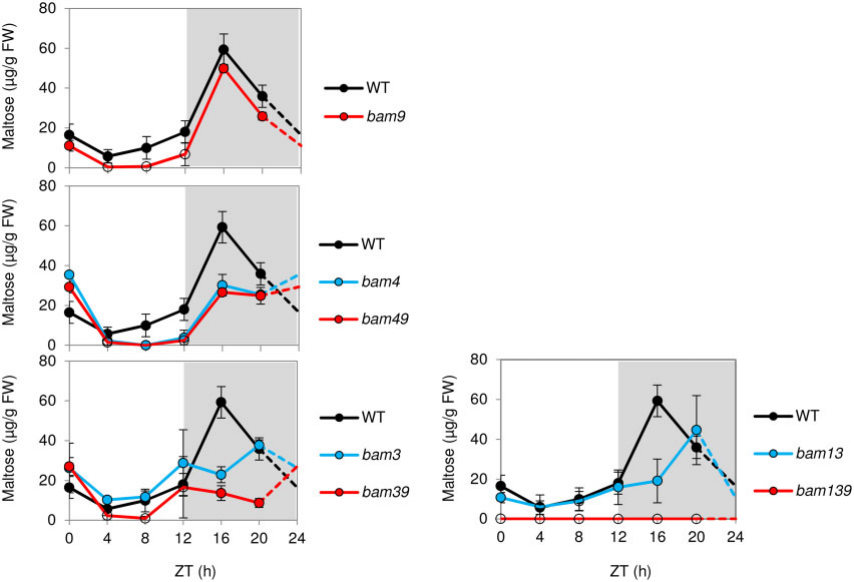
Effect of *bam9*-1 mutation on the maltose content in different genetic backgrounds. Rosettes of 23-d-old Arabidopsis plants of the indicated genotypes, grown in 12-h d/12-h night regime were harvested at different time points throughout the diel cycle. Maltose was measured using high-performance anion exchange chromatography with pulsed amperometric detection (HPAEC-PAD; *n* = 3 ± se). The same data for the WT are shown in each panel. In several genotypes, one or more of the replicate samples was below the limit of detection at one or more time point (note that repeat analyses with more concentrated extracts confirmed that small amounts of maltose are present—see text). In this case, unfilled symbols are used. ZT24 samples were not measured; dashed lines are used to complete the pattern over the diel cycle.

We quantified maltose in a subset of the mutants, given that it is a key intermediate of the starch degradation pathway (Niittylä et al., 2004; Weise et al., 2005). In this experiment, maltose could be readily detected at night, although daytime levels were sometimes close to or below the level of detection. In the *bam9* mutant, maltose levels were generally similar to or slightly lower than in the WT, whereas nighttime maltose levels were lower in the *bam3*, *bam4*, and *bam1bam3*, as previously reported (Figure  7). In *bam4bam9*, maltose levels were comparable to *bam4*, but in *bam3bam9*, the nighttime levels were lower than in *bam3*. In *bam1bam3bam9*, maltose levels were below the level of detection throughout the diel cycle. However, a repeated experiment in which more concentrated extracts were used confirmed that small amounts of maltose were present (21 ± 2 µg g^−1^ FW at the end of the night, compared with 37 ± 1 µg g^−^^1^ FW in *bam1bam3* and 57 ± 1 µg g^−1^ FW in the WT).

We crossed *bam9* mutant with the *lsf1* mutant. It has been shown that LSF1 interacts with BAM1 and BAM3 (Schreier et al., 2019) and is proposed to promote starch degradation by aiding the association of these BAMs with the starch granule surface. Starch degradation in *lsf1* mutants is reduced and starch accumulates to elevated levels. Again, loss of BAM9 enhanced this phenotype and the *lsf1bam9* double mutants had higher levels of leaf starch than the *lsf1* mutant (Supplemental Figure S5).

### Overexpression of BAM9 leads to premature exhaustion of starch reserves but cannot rescue *bam4* mutant phenotype

**Figure 8:**
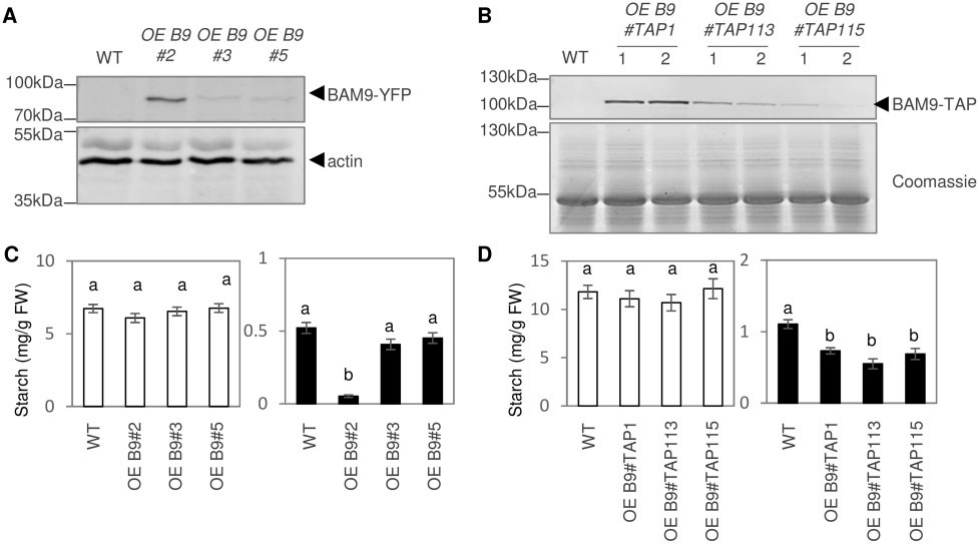
Strong overexpression of *BAM9* accelerates starch breakdown. A, BAM9-YFP protein abundance in the different *BAM9* overexpressing lines. An anti-YFP antibody was used to detect the BAM9-YFP proteins. An anti-actin antibody was used to detect actin, serving as a loading control. B, BAM9-TAP protein abundance in the different *BAM9* overexpressing lines. An anti-Myc antibody was used to detect the BAM9-TAP proteins. In each case, lanes 1 and 2 are biological replicates. Coomassie staining of the blot was used as loading control. C, Starch content at the end of the day (white bars) and end of the night (black bars) of the OE BAM9-YFP lines. *N* = 20 ± se from four independent experiments. Different letters indicate statistically significant difference (Tukey’s honestly significant difference [HSD], *P* < 0.05). D, Starch content at the end of the day (white bars) and end of the night (black bars) of the OE BAM9-TAP lines. *N *= 12 ± se from two independent experiments. Different letters indicate statistically significant differences (Tukey’s HSD, *P* < 0.05).

To further investigate the importance of BAM9 in regulating leaf starch metabolism, we measured the starch content in transgenic plants constitutively expressing either the BAM9–YFP fusion protein (Figure  2B) or BAM9 fused to a C-terminal TAP tag, under control of the ubiquitin and the 35S promoter, respectively. Several independent mono-insertional T3 lines were obtained for each construct (Figure  8). Lines expressed each protein to different extents, judging from immunoblots using the anti-YFP and anti-myc antibodies (Figure  8, A and B). We measured BAM activity in crude extracts in the line overexpressing BAM9–YFP to the highest extent (OE B9 #2) but the activity (2.43 ± 0.05 nmol min^−1^ mg^−1^ FW) was not substantially different from the WT. Interestingly, in most BAM9-expressing lines, there was a decrease in starch content at the end of the night, while starch content at the end of day remained largely un-affected (Figure  8, C and D). Thus, while loss of BAM9 conditionally leads to excess starch accumulation in leaves, its overexpression causes increased utilization of starch reserves at night. Starch also accumulates in the columella cells of the root tip, and we investigated the levels using light microscopy of cleared, iodine-stained roots (Supplemental Figure S6). No difference was observed between the WT and the *bam9* mutant but the line overexpressing BAM9–YFP to the highest extent (OE B9 #2) had less starch in its root-tip columella cells, similar to the observation in leaves.

**Figure 9:**
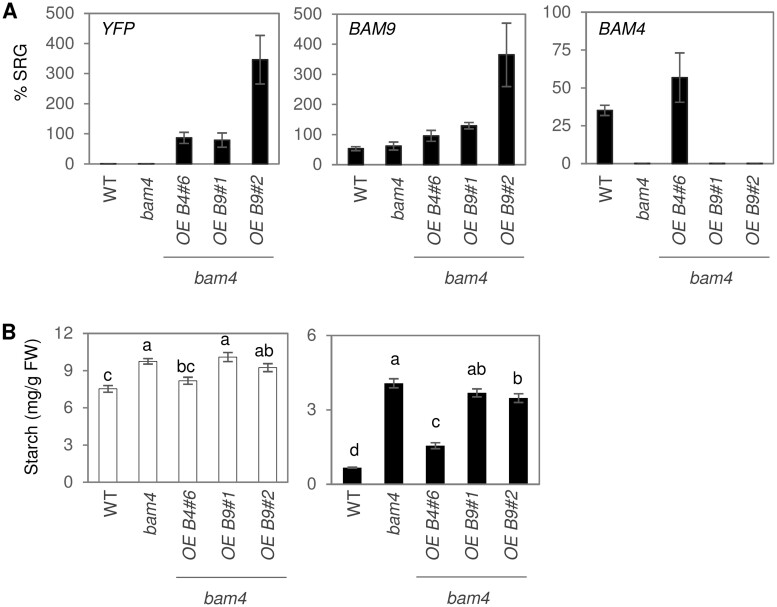
*BAM9* overexpression cannot complement *bam4* starch-excess phenotype. A, RT-qPCR was performed with specific primers for YFP. Two housekeeping genes were used to normalize expression (SRG, Synthetic Relative Gene: *RHIP* and *YSL8*). *N* = 3 ± SE. B, Starch content at the end of the day (white bars, left panel) and end of the night (black bars, right panel). *N* = 15 ± se from three different experiments. Different letters indicate statistically significant differences (Tukey’s HSD, *P* < 0.05).

We also overexpressed BAM9–YFP in the *bam4* mutant to see whether it was able to rescue the starch-excess phenotype of that line. In parallel, we transformed *bam4* mutant with a similar construct encoding BAM4–YFP. We used RT-qPCR to determine the level of expression of each construct and compared transformed lines with similar expression of either BAM4–YFP or BAM9–YFP (Figure  9A). While BAM4–YFP expression complemented the *bam4* mutant phenotype, BAM9–YFP expression (in the line *bam4*-OE9#1) did not (Figure  9B). We analyzed a second BAM9–YFP expressing line (*bam4*-OE9#2) with considerably stronger expression, but this line still had a starch-excess phenotype similar to *bam4*. This failure of BAM9 to rescue the *bam4* phenotype suggests that BAM4 and BAM9 functions are nonredundant.

## Discussion

Our results establish BAM9 as a regulator of transitory starch metabolism in Arabidopsis leaf chloroplasts. Although BAM9 acts alongside the homologous protein, BAM4, the functions of the two proteins appear to differ.

### BAM9 is a catalytically inactive BAM

Our reporter constructs show that BAM9 is expressed in both the shoots and the roots, with signals in leaf, particularly from the vasculature, and from the root tips. However, expression levels are relatively low, and very few BAM9 peptides are found in proteomic databases, suggesting that the protein is not abundant. During the diel cycle, *BAM9* expression in the leaves increased at night, peaking toward dawn, as was reported previously (Smith et al., 2004). Analysis of a wide range of transcriptome datasets using Genevestigator (Hruz et al., 2008) revealed that *BAM9* expression is responsive to environmental cues, being regulated by carbon (repressed by sugars), by the circadian clock, and by light signaling (e.g. Flis et al., 2016). In contrast, the expression of *BAM4* is relatively stable, with little response to environmental cues (Supplemental Figure S7).

Although bioinformatic predictions did not suggest that BAM9 is a chloroplastic protein, our results show that it is plastidial, and that it has a transit peptide located within the first 71 amino acid of its N-terminus. However, the structure predictions that BAM9 is a nonenzymatic protein appear to be correct, like for BAM4 (Fulton et al., 2008). Modeling of these proteins on the crystal structure of sweet potato BAM (Vajravijayan et al., 2018) revealed several features required for enzymatic catalysis, which are highly conserved and similarly oriented in the active sited of other active BAMs, are substituted or missing in BAM9, and that there is poor overall conservation of the active site among BAM9 orthologs. In active BAMs, glucan hydrolysis is catalyzed by two glutamic acid residues that act as general acid–base catalysts (Mikami et al., 1994; Kang et al., 2005). When substrate bound, one glutamic acid donates a proton to the glycosidic oxygen while the other activates a reactant water molecule. A conserved threonine stabilizes the deprotonated glutamic acid and the substrate via hydrogen bonds (Kang et al., 2005; Vajravijayan et al., 2018). Mutation of any of these residues drastically decreases enzyme activity (Kang et al., 2004, 2005), and both BAM4 and BAM9 are substituted in the second glutamic acid and the stabilizing threonine. Furthermore, the flexible loop that changes conformation to anchor the glucan substrate (Mikami et al., 1993, 1994) is also missing in BAM9. It is therefore unsurprising that we were not able to measure activity with recombinant BAM9, using either *p*-nitrophenyl β-maltotrioside or soluble starch as substrates. Similarly, it is unsurprising that there was no difference to the WT in total BAM activity in crude extracts of *bam9* mutant or the line overexpressing BAM9–YFP. Interestingly, while making these measurements, we observed that the active BAM BAM3 acted on the chromogenic substrate *p*-nitrophenyl β-maltotrioside (Betamyl-3), with a much lower specific activity than against soluble starch (Supplemental Figure S2). We suggest that this may be because the maltotriosyl chain is too short to be efficiently bound in the active site. Furthermore, we were unsuccessful in using the Betamyl-3 reagent to measure BAM activity in crude extracts of leaves.

Despite being catalytically inactive, BAM4 can reportedly still bind to starch (Fulton et al., 2008; Li et al., 2009). For BAM9, however, binding to starch granules in vitro was not robustly observed, although we note that there was some variation between experimental replicates (Supplemental Figure S3). Consistent with this, we did not detect an obvious granule localization for the YFP-tagged BAM9 protein in vivo, as was seen for other starch-binding proteins such as GRANULE-BOUND STARCH SYNTHASE (Seung et al., 2015) or EARLY STARVATION 1 (Feike et al., 2016). Thus, if BAM9 can associate with starch, it is with low affinity. Active BAMs can obviously bind a glucan chain via their functional substrate binding site, and BAMs found in some bacteria possess distinct CBMs (Janeček et al., 2019). Plant BAMs do not contain CBMs, although it was recently demonstrated that BAM1 and BAM3 are assisted in binding the starch granule through interaction with the CBM-containing scaffolding protein, LIKE SEX4 1 (Schreier et al., 2019). How BAM4 binds starch, and the functional significance of this ability, remain to be clarified. Binding could be mediated by surface-binding sites, which are difficult to predict using bioinformatics, but have been identified in numerous starch metabolizing enzymes (Wilkens et al., 2018).

### BAM9 positively regulates starch breakdown

Our data show that, despite its lack of enzymatic activity, BAM9 is an important player in Arabidopsis leaf starch metabolism. Like its nonenzymatic homolog BAM4, the role of BAM9 appears to be as a positive regulator of starch degradation, but unlike BAM4, its role is conditional. Constitutive overexpression of BAM9, either as a YFP-tagged or a TAP-tagged fusion protein in WT plants, resulted in lowered starch contents at the end of the night in leaves, and lower starch content in root tips, indicative of accelerated starch mobilization relative to the WT. Although plants lacking BAM9 grew similarly to the WT and had normal starch contents throughout the diel cycle, its loss in other mutant backgrounds with existing starch-excess phenotypes made these phenotypes significantly worse and impacted on plant growth.

There are other examples of conditional phenotypes in the process of starch degradation. Individually, the loss of the BAM BAM1, the α-amylase AMY3, or the debranching enzyme LDA do not affect starch metabolism in the leaf mesophyll, while their loss of in other mutant backgrounds does (Yu et al., 2005; Delatte et al., 2006; Fulton et al., 2008; Kötting et al., 2009; Streb et al., 2012; Seung et al., 2016). This can be explained by redundancy in the network of reactions by which the starch glucans are hydrolysed (i.e. the loss of BAM1 can be compensated by the presence of BAM3; the loss of AMY3 can be compensated by the presence of BAMs and debranching enzymes, etc.). However, the conditional nature of the BAM9 phenotype is more difficult to explain. One possibility would be partial redundancy between BAM4 and BAM9, with BAM4 able to fully compensate for the loss of BAM9. The fact that the *bam4bam9* double mutant’s phenotype was more severe than that of *bam4* would be consistent with this idea, but other results are not. For instance, the loss of BAM9 in the *bam3* mutant or in the *bam1bam3* double mutant worsened their starch-excess phenotypes despite the presence of BAM4. Indeed, the phenotype of the *bam1bam3bam9* mutant was extreme, with a very high starch content relative to the already strongly affected *bam1bam3* double mutant, and even lower nighttime maltose levels. These results also suggest that regulation of starch degradation by BAM9 involves proteins other than the active BAMs, BAM1 and BAM3. The loss of BAM9 also worsened the starch-excess phenotype of the *lsf1* mutant. This result makes sense, since the LSF1 protein is proposed to facilitate starch breakdown by BAM1 and BAM3 by helping them to associate with the starch granule surface (Schreier et al., 2019).

### BAM9 and BAM4 differ in expression and function

Despite the relatedness and apparent similarities between BAM9 and BAM4, there are interesting and important differences between the two. First, the expression pattern of BAM9 and BAM4 differ: BAM4 expresses predominantly in the vasculature (Francisco et al., 2010), while BAM9 is expressed both in the vasculature and in mesophyll cells. Second, while BAM4–YFP could rescue the starch-excess phenotype of the *bam4* mutant, a comparable degree of BAM9–YFP expression could not. Even when overexpressed at considerably higher levels, BAM9 could not compensate for the loss of BAM4, further pointing toward these two proteins having distinct functions. One possibility is that *BAM9* may serve as an inducible regulator of starch degradation, since its expression seems to be influenced by the circadian clock and by environmental conditions, including abiotic stresses like salinity and drought that can trigger starch degradation (Prasch et al., 2015; Flis et al., 2016; Horrer et al., 2016; Thalmann et al., 2016; Ghorbani et al., 2019). Indeed, we observed that BAM9 is expressed in stomatal guard cells, where BAM1- and AMY3-mediated starch degradation release osmolytes and respiratory substrates to drive stomata opening (Horrer et al., 2016).

Importantly, BAM9 is conserved in land plants, including monocotyledons, Fabaceae, Lamiids, and tree species like Salicaceae and citrus (Thalmann et al., 2019). In contrast, BAM4 appears to have been lost in many families. Thus, BAM9 could represent a widespread regulator of starch metabolism. For instance, in the Salicaceae, starch remobilization occurs during winter to ensure resistance to cold and during spring to ensure vegetative growth (Ashworth et al., 1993; Sauter and van Cleve, 1994; Von Fircks and Sennerby-Forsse, 1998; Ogren, 1999; Elle and Sauter, 2000; Palonen et al., 2008). In poplar—a member of the Salicaceae—*BAM9* is not only expressed both in leaves, following a circadian rhythm, but also in buds, and is induced in response to cold stress (Geisler-Lee et al., 2006; Hoffman et al., 2010). In banana, two *BAM9* paralogs are present (although mis-annotated as BAM4 in some studies). Their expression increases during fruit ripening, when starch is being broken down into sugars, but is severely repressed by cold storage, when starch degradation is inhibited (Miao et al., 2016; Xiao et al., 2018; Song et al., 2019).

In conclusion, our data indicate that the BAM-like protein BAM9 is a conserved regulator of starch degradation. Further genetic and biochemical analyses are required to better understand the molecular function of the BAM9 protein (e.g. through the investigation of interacting proteins) and to evaluate homologous genes as potential targets to improve starch traits in crop species.

## Materials and methods

### Plant material

Arabidopsis *(A.* *thaliana)* plants (Col-0 ecotype) were grown in a climate-controlled chamber with a constant temperature of 20°C, 60% relative humidity, with a 12-h photoperiod with a uniform light intensity of 150 µmol photons m^−2^ s^−1^. Two mutant alleles of At5g18670 (*BAM9*, also called* BMY3*) were characterized in this work: *bam9-1* (SALK_021635), and *bam9-2* (GABI_722D02). The following mutants characterized by Fulton et al. (2008) or Comparot-Moss et al. (2010) were also used to generate crosses: *bam1-1*, SALK_039895; *bam3-1*, CS92461; *bam4-1*, SALK_037355 and *lsf1-2*, SALK_053285.

### RNA extraction and RT-qPCR

Total RNA was isolated from individual rosettes using GENEzol extraction buffer according to the manufacturer guidelines (Geneaid Biotech, New Taipei City, Taiwan). Genomic DNA contamination was removed using DNase I (Roche, Basel, Switzerland). First-strand cDNAs were synthesized from 1 µg RNA using oligo(dT)18 primers and the RevertAid First Strand cDNA Synthesis Kit (Thermo Fisher Scientific, Waltham, MA, USA). Resultant cDNA was diluted 25-fold and 2.5 µL was used in a 10 µL reaction of KAPA SYBR FAST qPCR Master Mix (2×) Universal (KAPA Biosystem, Wilmington MA, USA). RT-PCR reactions were performed with BAM9 gene-specific primers and control primers for the housekeeping gene ACTIN2. Real-time quantitative PCR reactions were performed using the LightCycler 480 II system (Roche). Efficiencies of each primer pair were similar. Primer sequences are listed in Supplemental Dataset S1. The results were standardized using two reference genes that were averaged by their geometric mean as a Synthetic Reference Gene, as described (Vandesompele et al., 2002).

### Cloning of expression vectors for plant transformation

For expression pattern analysis, a fragment of the *BAM9* gene was cloned in frame with the *GUS* reporter gene in the pGWB533 plasmid (Nakagawa et al., 2007). The *BAM9* gene fragment started 2.3-kb upstream of the start codon and included the part of the gene encoding the first 204 amino acids of the protein, resulting in a translational fusion. Expression patterns in several T1 plants were compared to select a line with strong, representative expression. Plants from the T3 generation were stained for GUS activity. For sub-cellular localization in Arabidopsis leaf mesophyll protoplasts, the *BAM9* CDS, lacking the stop codon, was cloned into pJJ461 (Lee et al., 2007). The first 213 nucleotides of the CDS, corresponding to a putative 71 amino acid chloroplastic transit peptide, was cloned separately into p2GWGF7. This resulted in sequences in frame with a C-terminal YFP tag and downstream of the 35S CaMV promoter in each case. Protoplasts were prepared from the leaf mesophyll of WT plants according to Fitzpatrick and Keegstra (2001). Transient expression was performed by polyethylene glycol-mediated transfection as described by Jin et al. (2001). For stable expression of YFP-fused protein in WT or *bam4* mutant plants, *BAM9* and *BAM4* CDS were amplified without stop codon from cDNA and cloned into pDONR221 using BP clonase (Invitrogen, Waltham, MA, USA). These plasmids were then recombined into pUBC-YFP-DEST using an LR clonase (Invitrogen), resulting in an in-frame fusion of the CDS with a C-terminal YFP tag, under control of ubiquitin promoter. For stable expression of TAP-tagged BAM9 protein in WT plants, the BAM9 CDS was cloned downstream of the 35S promoter and in frame with a C-terminal myc tag by recombination of the aforementioned BAM9-pDONR221 into pYL436 (Rubio et al., 2005). All primers used for cloning are listed in Supplemental Table S1.

### Confocal microscopy

Microscopy imaging was performed using a Zeiss LSM 780 confocal microscope (Carl Zeiss, Feldbach, Switzerland). An argon laser was used for excitation (at 514 nm). Emission was captured between 462 and 500 nm for YFP and between 662 and 721 nm for chlorophyll autofluorescence.

### Protein structure modeling

Structure predictions for AtBAM3, AtABM4, and AtBAM9 were performed using SWISS-MODEL. The crystallized BAM from sweet potato (Vajravijayan et al., 2018) was chosen as the template based on QMEAN values. We used the resolved crystal structure bound to maltotetraose (5WQU_1). Graphic modifications were done using PYMOL2 software. Amino acid conservation was determined based on multiple protein alignment done with Clustal Omega. For each protein, we used sequences of several orthologs belonging to angiosperms, basal angiosperm, and gymnosperms that were identified by Thalmann et al. (2019). Accession numbers are given in Supplemental Dataset S3.

### Recombinant protein expression in *E.coli*

The CDS of *BAM9*, lacking the first 71 amino acids, was amplified either with EcoRI and XbaI restriction sites from Arabidopsis cDNA and cloned into the pProEX Htb vector, or with EcoRI and SalI restriction sites and cloned into the pET28a+ expression vector, resulting in a protein with a 6× His-tag at the N-terminal and C-terminal ends of the protein, respectively. Similarly, we cloned BAM3 CDS lacking its predicted 49 amino acid chloroplast transit peptide in the same vector. All cloning primer sequences are given in Supplemental Dataset S1.

For expression in *E. coli* Arctic Express (DE3) RIL, transformed cells were grown at 30°C for 3 h prior induction by addition of 1 mM isopropyl β-D-1-thiogalactopyranoside. Cells were then incubated for 18.5 h at 13°C, and subsequently harvested by centrifugation (15 min, 3,000*g*, 4°C). Cells were resuspended in 50 mM Tris–HCl, pH 7.5, 300 mM NaCl, 40 mM imidazole, 1 mg/mL Lysozyme, 1× Protease Inhibitor Cocktail (Roche), 2 mM dithiothreitol (DTT), and lysed using a microfluidizer. Lysates were cleared by centrifugation (10 min, 20,000 *g*, 4°C) and filtering through a 0.45 μm mesh. Cleared lysates were loaded on HisTrap HP 1 mL columns (using an Aekta Pure 25 system; Cytiva, Opfikon, Switzerland), washed with 50 mM Tris–HCl pH 7.5, 300 mM NaCl, 40 mM imidazole, 2 mM DTT, followed by the same medium with 63 mM imidazole. Bound proteins were eluted using the same medium with a linear gradient of imidazole concentration increasing up to 500 mM. Eluted proteins were buffer-exchanged into 20 mM Tris–HCl pH 7.5, 50 mM NaCl, 2 mM DTT using NAP-25 columns, concentrated using Vivaspin 2—PES concentrators with a molecular weight cut-off of 50 kDa, and finally buffer-exchanged into 10 mM MOPS-NaOH pH 7.5, 50 mM NaCl, 2 mM DTT, aliquoted and snap frozen in liquid N_2_ prior to analysis.

### Far-UV CD spectroscopy

Far-UV CD spectra of recombinant BAM3 and BAM9 proteins were acquired at 25°C, a protein concentration of 0.2 μg/μL in 10 mM MOPS-NaOH pH 7.5, 50 mM NaCl, 2 mM DTT and using a cuvette with 1 mm path length. Data acquisition was done using a Jasco Spectropolarimeter (Jasco, Easton, USA) between 260 and 200 nm, using a data pitch of 0.5 nm and a band width of 2 nm. Recorded protein spectra were buffer-corrected and normalized to mean residue ellipticity according to Schmid (2005).

### Starch binding experiments

Starch binding capacity of BAM9 was assayed in vitro as described by Seung et al. (2015). In brief, 1 μg of recombinant protein was incubated in a binding medium containing 50 mM MOPS-NaOH pH 7.5, 2 mM MgCl_2_, 0.1% [w/v] bovine serum albumin (BSA), 1 mM DTT, 0.01% [v/v] Triton X-100, 4 mM NaCl, with 30 mg of hydrated maize starch (Sigma-Aldrich, St Louis, MO, USA), or Sephadex G10 resin (Sigma-Aldrich) as negative control. After incubation on spinning wheel, the supernatant was collected, the pellet washed 3 times with the same buffer, and the pellet-bound proteins eluted with incubation medium, to which 2% (w/v) sodium dodecyl sulfate (SDS) was added.

### BAM activity

BAM activities of recombinant BAM9 and BAM3 proteins were assayed using the Betamyl-3 assay kit (Megazyme, Bray, Ireland) or by performing in-vitro digestion of soluble starch (from potato; Sigma-Aldrich). For activity on Betamyl-3, recombinant proteins were assayed in final concentrations of 25 mM MOPS-NaOH pH 7.5, 25 mM NaCl, 1 mM DTT, 0.05% [w/v] BSA together with Betamyl-3 substrate at 25°C for either 10 or 20 min, as indicated. Complete deprotonation of the released 4-nitrophenol was achieved by raising the pH of the reactions to 8.5 by addition of 1 M Tris–HCl pH 8.5. Absorbance of the released 4-nitrophenol was subsequently measured at 400 nm using a Tecan M1000 plate reader. For activity measurements using soluble starch, the substrate was solubilized by boiling for 15 min, cooled to 25°C, and subsequently incubated with recombinant protein for 15, 30, or 60 min, as indicated, at 25°C. Reactions contained 25 mM MOPS-NaOH pH 7, 10% [v/v] ethylene glycol, 5 mM ethylenediaminetetraacetic acid (EDTA, 5 mM DTT, 15 mg/mL soluble potato starch, and the indicated recombinant protein amounts. Reactions were stopped by boiling for 15 min at 100°C. For T0 time points, enzymes were inactivated by boiling for 15 min before addition of the starch substrate. Released maltose was separated by HPAEC on a Carbopac PA200 column and detected with PAD using a BioLC (Dionex, Sunnyvale, CA, USA).

To assay BAM activity in crude protein extracts, leaves were homogenized in extraction medium containing 50 mM MOPS, pH 7, 20% (v/v) ethylene glycol, 10 mM DTT, 10 mM EDTA, and the homogenate clarified by centrifugation (10 min, 20,000 *g*, 4°C). BAM activity in the supernatant was assayed against soluble starch, as described above.

### Carbohydrate quantification

Entire rosettes of individual plants (23–25 d old) were frozen and pulverized using a Mixer Mill (Retsch, Haan, Germany). Starch and soluble sugars were extracted from the frozen powder and quantified as described in Hostettler et al. (2011). Briefly, after perchloric acid extraction, starch in the insoluble fraction was converted into glucose by treatment with BAM and amyloglucosidase. The released glucose was then quantified with an enzymatic assay. To quantify maltose, the soluble fraction was neutralized and neutral compounds were obtained by passage through sequential Dowex 50W and Dowex 1 columns (Sigma, St Louis, MO, USA). Maltose was determined by HPAEC-PAD.

For the microscopic analysis of starch in root tips, Arabidopsis seeds were surface-sterilized with 70% (v/v) ethanol and sown on agar plates containing half-strength Murashige and Skoog medium. Seedlings were harvested at midday (ZT7) after 13 d of growth in a 12-h light/12-h dark cycle. Root tips were immersed in Lugol’s iodine solution (Sigma) for 30 s, then immediately placed on a slide containing Hertwig’s Solution (acidified chloral hydrate–glycerol solution). Samples were examined with a Leica DFC 7000T microscope with a bright field filter (Leica Microsystems, Heerbrugg, Switzerland).

### Protein extraction and immunoblotting

Total proteins were extracted from rosettes homogenized in 50 mM Tris–HCl, pH 8, 150 mM NaCl, 1% (v/v) Triton X-100, 1 mM DTT, 1× Complete Protease Inhibitor EDTA-free Mixture (Roche). Equal amounts of soluble proteins were separated by SDS-polyacrylamide gel electrophoresis (SDS–PAGE) and subject to immunoblotting. For stable lines expressing YFP-tagged proteins, anti-GFP/YFP (rabbit, ab290; Abcam, Cambridge, UK) and anti-plant actin (mouse, A0480; Sigma-Aldrich) were used. For immunoblotting of BAM9, we used antibodies raised in rabbits (Eurogentec, Seraing, Belgium) against the recombinant purified protein. For immunoblotting of BAM3, we used antibodies described previously (Fulton et al., 2008). Proteins were detected using near-infrared fluorescence from IRDye secondary antibodies (anti-rabbit and/or anti-mouse) and an Odyssey CLx detection system (Li-Cor, Lincoln, NE, USA). For the stable BAM9-TAP lines, an anti-myc (mouse, M4439; Sigma-Aldrich) antibody was used to detect the proteins followed by colorimetric detection.

## Accession numbers

Please refer to Supplemental Dataset S2 and Supplemental Dataset S3.

## Supplemental data

The following materials are available in the online version of this article.

**Supplemental Figure S1.** *BAM9* expression over the diel cycle.

**Supplemental Figure S2.** Activity of recombinant BAM3 and BAM9 proteins when incubated with soluble starch.

**Supplemental Figure S3.** Binding of recombinant BAM3 and BAM9 proteins to starch granules.

**Supplemental Figure S4.** Analysis of the starch contents of multiple mutants produced using the *bam9*-2 mutant allele.

**Supplemental Figure S5.** Analysis of the starch contents of the *bam9 lsf1* double mutant.

**Supplemental Figure S6.** Starch in the root tips of WT, *bam9* mutant, and *BAM9* over-expressing plants.

**Supplemental Figure S7.** Response of *BAM9* and *BAM4* gene transcription upon environmental perturbations.

**Supplemental Dataset S1.** List of the oligonucleotide primers used for the different experiments of this study.

**Supplemental Dataset S2.** List of the accession numbers of the BAM sequences used for structural modeling.

**Supplemental Dataset S3.** List of the accession numbers of the BAM sequences used for the residue conservation analysis.

## Supplementary Material

kiab468_Supplementary_Data

## Notes

S.C.Z. conceived the original research plan and supervised the experiments; S.C.Z., L.C.D., and S.K.L. designed the experiments and analyzed the data; L.C.D., S.K.L., M.R.A., E.M.S., M.F.S., and M.A.T. performed the molecular genetic and biochemical experiments. E.B. and K.S. provided L.C.D. with technical assistance. L.C.D. and S.C.Z. wrote the article with contributions of all the authors; S.C.Z. agrees to serve as the author responsible for contact and ensures communication.

The author responsible for distribution of materials integral to the findings presented in this article in accordance with the policy described in the Instructions for Authors (https://academic.oup.com/plphys/pages/general-instructions) is Samuel C. Zeeman (szeeman@ethz.ch).

## References

[kiab468-B1] Ashworth EN , Stirm VE, Volenec JJ (1993) Seasonal variations in soluble sugars and starch within woody stems of *Cornus sericea* L. Tree Physiol 13: 379–388 14969993 10.1093/treephys/13.4.379

[kiab468-B2] Comparot-Moss S , Kötting O, Stettler M, Edner C, Graf A, Weise SE, Streb S, Lue WL, MacLean D, Mahlow S, et al (2010) A putative phosphatase, LSF1, is required for normal starch turnover in Arabidopsis leaves. Plant Physiol 152: 685–697 20018601 10.1104/pp.109.148981 PMC2815883

[kiab468-B3] Delatte T , Umhang M, Trevisan M, Eicke S, Thorneycroft D, Smith SM, Zeeman SC (2006) Evidence for distinct mechanisms of starch granule breakdown in plants. J Biol Chem 17: 12050–12059 10.1074/jbc.M513661200 16495218

[kiab468-B4] Dong S , Zhang J, Beckles DM (2018) A pivotal role for starch in the reconfiguration of ^14^C-partitioning and allocation in *Arabidopsis thaliana* under short-term abiotic stress. Sci Rep 8: 9314 29915332 10.1038/s41598-018-27610-y PMC6006365

[kiab468-B5] Elle D , Sauter JJ (2000) Seasonal changes of activity of a starch granule bound endoamylase and of a starch phosphorylase in poplar wood (*Populus* × *canadensis* Moench <robusta>) and their possible regulation by temperature and phytohormones. J Plant Physiol 156: 731–740

[kiab468-B6] Emanuelsson O , Nielsen H, Heijne GV (1999) ChloroP, a neural network-based method for predicting chloroplast transit peptides and their cleavage sites. Protein Sci 8: 978–984 10338008 10.1110/ps.8.5.978 PMC2144330

[kiab468-B7] Feike D , Seung D, Graf A, Bischof S, Ellick T, Coiro M, Soyk S, Eicke S, Mettler-Altmann T, Lu KJ, et al (2016) The starch granule-associated protein EARLY STARVATION1 Is required for the control of starch degradation in *Arabidopsis thaliana* leaves. Plant Cell 28: 1472–1489 27207856 10.1105/tpc.16.00011 PMC4944407

[kiab468-B8] Fernandez O , Ishihara H, George GM, Mengin V, Flis A, Sumner D, Arrivault S, Feil R, Lunn JE, Zeeman SC, et al (2017) Leaf starch turnover occurs in long days and in falling light at the end of the day. Plant Physiol 174: 2199–2212 28663333 10.1104/pp.17.00601 PMC5543966

[kiab468-B9] Fitzpatrick LM , Keegstra K (2001) A method for isolating a high yield of Arabidopsis chloroplasts capable of efficient import of precursor proteins. Plant J 27: 59–65 11489183 10.1046/j.0960-7412.2001.01061.x

[kiab468-B10] Flis A , Sulpice R, Seaton DD, Ivakov AA, Liput M, Abel C, Millar AJ, Stitt M (2016) Photoperiod-dependent changes in the phase of core clock transcripts and global transcriptional outputs at dawn and dusk in Arabidopsis. Plant Cell Environ 39: 1955–1981 27075884 10.1111/pce.12754

[kiab468-B11] Flütsch S , Wang Y, Takemiya A, Vialet-Chabrand SRM, Klejchová M, Nigro A, Hills A, Lawson T, Blatt MR, Santelia D (2020) Guard cell starch degradation yields glucose for rapid stomatal opening in Arabidopsis. Plant Cell 32: 2325–2344 32354788 10.1105/tpc.18.00802 PMC7346545

[kiab468-B12] Francisco P , Li J, Smith SM (2010) The gene encoding the catalytically inactive β-amylase BAM4 involved in starch breakdown in Arabidopsis leaves is expressed preferentially in vascular tissues in source and sink organs. J Plant Physiol 167: 890–895 20153546 10.1016/j.jplph.2010.01.006

[kiab468-B13] Fulton DC , Stettler M, Mettler T, Vaughan CK, Li J, Francisco P, Gil M, Reinhold H, Eicke S, Messerli G, et al (2008) β-AMYLASE4, a noncatalytic protein required for starch breakdown, acts upstream of three active β-amylases in Arabidopsis chloroplasts. Plant Cell 20: 1040–1058 18390594 10.1105/tpc.107.056507 PMC2390740

[kiab468-B14] Geisler-Lee J , Geisler M, Coutinho PM, Segerman B, Nishikubo N, Takahashi J, Aspeborg H, Djerbi S, Master E, Andersson-Gunnerås S, et al (2006) Poplar carbohydrate-active enzymes. Gene identification and expression analyses. Plant Physiol 140: 946–962 16415215 10.1104/pp.105.072652 PMC1400564

[kiab468-B15] Ghorbani R , Alemzadeh A, Razi H (2019) Microarray analysis of transcriptional responses to salt and drought stress in *Arabidopsis thaliana*. Heliyon 5: e02614 31844689 10.1016/j.heliyon.2019.e02614 PMC6895597

[kiab468-B16] Gibon Y , Bläsing OE, Palacios‐Rojas N, Pankovic D, Hendriks JHM, Fisahn J, Höhne M, Günther M, Stitt M (2004) Adjustment of diurnal starch turnover to short days: depletion of sugar during the night leads to a temporary inhibition of carbohydrate utilization, accumulation of sugars and post‐translational activation of ADP‐glucose pyrophosphorylase in the following light period. Plant J 39: 847–862 15341628 10.1111/j.1365-313X.2004.02173.x

[kiab468-B17] Graf A , Schlereth A, Stitt M, Smith AM (2010) Circadian control of carbohydrate availability for growth in Arabidopsis plants at night. Proc Natl Acad Sci USA 107: 9458–9463 20439704 10.1073/pnas.0914299107 PMC2889127

[kiab468-B18] Hejazi M , Fettke J, Paris O, Steup M (2009) The two plastidial starch-related dikinases sequentially phosphorylate glucosyl residues at the surface of both the A- and B-Type allomorphs of crystallized maltodextrins but the mode of action differs. Plant Physiol 150: 962–976 19395406 10.1104/pp.109.138750 PMC2689988

[kiab468-B19] Hoffman DE , Jonsson P, Bylesjö M, Trygg J, Antti H, Eriksson ME, Moritz T (2010) Changes in diurnal patterns within the Populus transcriptome and metabolome in response to photoperiod variation: changes in diurnal patterns. Plant Cell Environ 33: 1298–1313 20302601 10.1111/j.1365-3040.2010.02148.x

[kiab468-B20] Hooper CM , Castleden I, Tanz SK, Aryamanesh N, Millar AH (2017) SUBA4: the interactive data analysis centre for Arabidopsis subcellular protein locations. Nucleic Acids Res 45**:** D1064–D1074 27899614 10.1093/nar/gkw1041 PMC5210537

[kiab468-B21] Horrer D , Flütsch S, Pazmino D, Matthews JSA, Thalmann M, Nigro A, Leonhardt N, Lawson T, Santelia D (2016) Blue light induces a distinct starch degradation pathway in guard cells for stomatal opening. Curr Biol 26: 362–370 26774787 10.1016/j.cub.2015.12.036

[kiab468-B22] Hostettler C , Kölling K, Santelia D, Streb S, Kötting O, Zeeman SC (2011) Analysis of starch metabolism in chloroplasts. Methods Mol Biol 775: 387–410 21863455 10.1007/978-1-61779-237-3_21

[kiab468-B23] Hruz T , Laule O, Szabo G, Wessendorp F, Bleuler S, Oertle L, Widmayer P, Gruissem W, Zimmermann P (2008) Genevestigator v3: a reference expression database for the meta-analysis of transcriptomes. Adv Bioinform 2008: 420747 10.1155/2008/420747 PMC2777001 19956698

[kiab468-B24] Janeček Š , Mareček F, MacGregor EA, Svensson B (2019) Starch-binding domains as CBM families–history, occurrence, structure, function and evolution. Biotechnol Adv 37: 107451 31536775 10.1016/j.biotechadv.2019.107451

[kiab468-B25] Jin JB , Kim YA, Kim SJ, Lee SH, Kim DH, Cheong GW, Hwang I (2001) A new dynamin-like protein, ADL6, is involved in trafficking from the trans-golgi network to the central vacuole in Arabidopsis. Plant Cell 13: 1511–1526 11449048 10.1105/TPC.000534 PMC139540

[kiab468-B26] Kang YN , Adachi M, Utsumi S, Mikami B (2004) The roles of Glu186 and Glu380 in the catalytic reaction of soybean β-amylase. J Mol Biol 339: 1129–1140 15178253 10.1016/j.jmb.2004.04.029

[kiab468-B27] Kang YN , Tanabe A, Adachi M, Utsumi S, Mikami B (2005) Structural analysis of threonine 342 mutants of soybean β-amylase: role of a conformational change of the inner loop in the catalytic mechanism. Biochemistry 44: 5106–5116 15794648 10.1021/bi0476580

[kiab468-B28] Kaplan F , Guy CL (2004) β-Amylase induction and the protective role of maltose during temperature shock. Plant Physiol 135: 1674–1684 15247404 10.1104/pp.104.040808 PMC519081

[kiab468-B29] Kaplan F , Guy CL (2005) RNA interference of Arabidopsis β-amylase8 prevents maltose accumulation upon cold shock and increases sensitivity of PSII photochemical efficiency to freezing stress. Plant J 44: 730–743 16297066 10.1111/j.1365-313X.2005.02565.x

[kiab468-B30] Kölling K , Thalmann M, Müller A, Jenny C, Zeeman SC (2015) Carbon partitioning in *Arabidopsis thaliana* is a dynamic process controlled by the plants metabolic status and its circadian clock. Plant Cell Environ 38: 1965–1979 25651812 10.1111/pce.12512 PMC4671261

[kiab468-B31] Kötting O , Santelia D, Edner C, Eicke S, Marthaler T, Gentry MS, Comparot-Moss S, Chen J, Smith AM, Steup M, et al (2009) STARCH-EXCESS4 is a laforin-like phosphoglucan phosphatase required for starch degradation in *Arabidopsis thaliana*. Plant Cell 21: 334–346 19141707 10.1105/tpc.108.064360 PMC2648081

[kiab468-B32] Laby RJ , Kim D, Gibson SI (2001) The *ram1* mutant of Arabidopsis exhibits severely decreased β-amylase activity. Plant Physiol 127: 1798–1807 11743123 PMC133583

[kiab468-B33] Lee SK , Hwang SK, Han M, Eom JS, Kang HG, Han Y, Choi SB, Cho MH, Bhoo SH, An G, et al (2007) Identification of the ADP-glucose pyrophosphorylase isoforms essential for starch synthesis in the leaf and seed endosperm of rice (*Oryza sativa* L.). Plant Mol Biol 65: 531–546 17406793 10.1007/s11103-007-9153-z

[kiab468-B34] Li J , Francisco P, Zhou W, Edner C, Steup M, Ritte G, Bond CS, Smith SM (2009) Catalytically-inactive β-amylase BAM4 required for starch breakdown in Arabidopsis leaves is a starch-binding-protein. Arch Biochem Biophys 489: 92–98 19664588 10.1016/j.abb.2009.07.024

[kiab468-B35] Miao H , Sun P, Miao Y, Liu J, Zhang J, Jia C, Wang J, Wang Z, Jin Z, Xu B (2016) Genome-wide identification and expression analysis of the β-amylase genes strongly associated with fruit development, ripening, and abiotic stress response in two banana cultivars. Front Agric Sci Eng 3: 346

[kiab468-B36] Mikami B , Hehre EJ, Sato M, Katsube Y, Hirose M, Morita Y, Sacchettini JC (1993) The 2.0-Å resolution structure of soybean β-amylase complexed with α-cyclodextrin. Biochemistry 32: 6836–6845 8334116 10.1021/bi00078a006

[kiab468-B37] Mikami B , Degano M, Hehre EJ, Sacchettini JC (1994) Crystal structures of soybean β-amylase reacted with β-maltose and maltal: active site components and their apparent roles in catalysis. Biochemistry 33: 7779–7787 8011643

[kiab468-B38] Monroe JD (2020) Involvement of five catalytically active Arabidopsis β-amylases in leaf starch metabolism and plant growth. Plant Direct 4: e00199 32072133 10.1002/pld3.199 PMC7011640

[kiab468-B39] Monroe JD , Storm AR (2018) The Arabidopsis β−amylase (BAM) gene family: diversity of form and function. Plant Sci 276: 163–170 30348315 10.1016/j.plantsci.2018.08.016

[kiab468-B40] Monroe JD , Breault JS, Pope LE, Torres CE, Gebrejesus TB, Berndsen CE, Storm AR (2017) Arabidopsis β-Amylase2 is a K^+^-requiring, catalytic tetramer with sigmoidal kinetics. Plant Physiol 175: 1525–1535 29066669 10.1104/pp.17.01506 PMC5717748

[kiab468-B41] Mugford ST , Fernandez O, Brinton J, Flis A, Krohn N, Encke B, Feil R, Sulpice R, Lunn JE, Stitt M, et al (2014) Regulatory properties of ADP-glucose pyrophosphorylase are required for adjustment of leaf starch synthesis in different photoperiods. Plant Physiol 166: 1733–1747 25293961 10.1104/pp.114.247759 PMC4256850

[kiab468-B42] Nakagawa T , Suzuki T, Murata S, Nakamura S, Hino T, Maeo K, Tabata R, Kawai T, Tanaka K, Niwa Y, et al (2007) Improved gateway binary vectors: high-performance vectors for creation of fusion constructs in transgenic analysis of plants. Biosci Biotechnol Biochem 71: 2095–2100 17690442 10.1271/bbb.70216

[kiab468-B43] Niittylä T , Messerli G, Trevisan M, Chen J, Smith AM, Zeeman SC (2004) A previously unknown maltose transporter essential for starch degradation in leaves. Science 303**:** 87–89 14704427 10.1126/science.1091811

[kiab468-B44] Niittylä T , Comparot-Moss S, Lue WL, Messerli G, Trevisan M, Seymour MDJ, Gatehouse JA, Villadsen D, Smith SM, Chen J, et al (2006) Similar protein phosphatases control starch metabolism in plants and glycogen metabolism in mammals. J Biol Chem 281: 11815–11818 16513634 10.1074/jbc.M600519200

[kiab468-B45] Ogren E (1999) Fall frost resistance in willows used for biomass production. II. Predictive relationships with sugar concentration and dry matter content. Tree Physiol 19: 755–760 12651315 10.1093/treephys/19.11.755

[kiab468-B46] Palonen P , Buszard D, Donnelly D (2008) Changes in carbohydrates and freezing tolerance during cold acclimation of red raspberry cultivars grown *in vitro* and *in vivo*. Physiol Plant 110: 393–401

[kiab468-B47] Prasch CM , Ott KV, Bauer H, Ache P, Hedrich R, Sonnewald U (2015) β-amylase1 mutant Arabidopsis plants show improved drought tolerance due to reduced starch breakdown in guard cells. J Exp Bot 66: 6059–6067 26139825 10.1093/jxb/erv323 PMC4566991

[kiab468-B48] Reinhold H , Soyk S, Simková K, Hostettler C, Marafino J, Mainiero S, Vaughan CK, Monroe JD, Zeeman SC (2011) β-amylase-like proteins function as transcription factors in Arabidopsis, controlling shoot growth and development. Plant Cell 23: 1391–1403 21487098 10.1105/tpc.110.081950 PMC3101533

[kiab468-B49] Rubio V , Shen Y, Saijo Y, Liu Y, Gusmaroli G, Dinesh‐Kumar SP, Deng XW (2005) An alternative tandem affinity purification strategy applied to Arabidopsis protein complex isolation. Plant J 41**:** 767–778 15703063 10.1111/j.1365-313X.2004.02328.x

[kiab468-B50] Santelia D , Kötting O, Seung D, Schubert M, Thalmann M, Bischof S, Meekins DA, Lutz A, Patron N, Gentry MS, et al (2011) The phosphoglucan phosphatase LIKE SEX FOUR2 dephosphorylates starch at the C3-position in Arabidopsis. Plant Cell 23**:** 4096–4111 22100529 10.1105/tpc.111.092155 PMC3246334

[kiab468-B51] Sauter JJ , van Cleve B (1994) Storage, mobilization and interrelations of starch, sugars, protein and fat in the ray storage tissue of poplar trees. Trees 8**:** 297–304

[kiab468-B52] Schmid F (2005) Spectroscopic techniques to study protein folding and stability. *In* Buchner J, Kiefhaber T, eds, Protein Folding Handbook, WILEY‐VCH Verlag GmbH & Co. KGaA, Weinheim, Germany, pp 22–44

[kiab468-B53] Schreier TB , Umhang M, Lee SK, Lue WL, Shen Z, Silver D, Graf A, Müller A, Eicke S, Stadler-Waibel M, et al (2019) LIKE SEX4 1 acts as a β-amylase-binding scaffold on starch granules during starch degradation. Plant Cell 31**:** 2169–2186 31266901 10.1105/tpc.19.00089 PMC6751131

[kiab468-B54] Scialdone A , Mugford ST, Feike D, Skeffington A, Borrill P, Graf A, Smith AM, Howard M (2013) Arabidopsis plants perform arithmetic division to prevent starvation at night. eLife 2**:** e00669 23805380 10.7554/eLife.00669 PMC3691572

[kiab468-B55] Seki M , Ohara T, Hearn TJ, Frank A, da Silva VCH, Caldana C, Webb ARR, Satake A (2017) Adjustment of the Arabidopsis circadian oscillator by sugar signalling dictates the regulation of starch metabolism. Sci Rep 7**:** 8305 28814797 10.1038/s41598-017-08325-y PMC5559614

[kiab468-B56] Seung D , Lu KJ, Stettler M, Streb S, Zeeman SC (2016) Degradation of glucan primers in the absence of starch synthase 4 disrupts starch granule initiation in Arabidopsis. J Biol Chem 291**:** 20718–20728 27458017 10.1074/jbc.M116.730648 PMC5034061

[kiab468-B57] Seung D , Soyk S, Coiro M, Maier BA, Eicke S, Zeeman SC (2015) PROTEIN TARGETING TO STARCH is required for localising GRANULE-BOUND STARCH SYNTHASE to starch granules and for normal amylose synthesis in Arabidopsis. PLoS Biol 13**:** e1002080 25710501 10.1371/journal.pbio.1002080 PMC4339375

[kiab468-B58] Smith AM , Zeeman SC (2020) Starch: a flexible, adaptable carbon store coupled to plant growth. Annu Rev Plant Biol 71**:** 217–245 32075407 10.1146/annurev-arplant-050718-100241

[kiab468-B59] Smith SM , Fulton DC, Chia T, Thorneycroft D, Chapple A, Dunstan H, Hylton C, Zeeman SC, Smith AM (2004) Diurnal changes in the transcriptome encoding enzymes of starch metabolism provide evidence for both transcriptional and posttranscriptional regulation of starch metabolism in Arabidopsis leaves. Plant Physiol 136: 2687–2699 15347792 10.1104/pp.104.044347 PMC523333

[kiab468-B60] Song Z , Qin J, Zheng Q, Ding X, Chen W, Lu W, Li X, Zhu X (2019) The Involvement of the banana F-Box protein MaEBF1 in regulating chilling-inhibited starch degradation through interaction with a MaNAC67-like protein. Biomolecules 9: 552 10.3390/biom9100552 PMC6843822 31575083

[kiab468-B61] Soyk S , Simková K, Zürcher E, Luginbühl L, Brand LH, Vaughan CK, Wanke D, Zeeman SC (2014) The enzyme-like domain of Arabidopsis nuclear β-amylases is critical for DNA sequence recognition and transcriptional activation. Plant Cell 26: 1746–1763 24748042 10.1105/tpc.114.123703 PMC4036583

[kiab468-B62] Sparla F , Costa A, Lo Schiavo F, Pupillo P, Trost P (2006) Redox regulation of a novel plastid-targeted β-amylase of Arabidopsis. Plant Physiol 141: 840–850 16698902 10.1104/pp.106.079186 PMC1489908

[kiab468-B63] Stettler M (2009) Maltose metabolism during starch breakdown in the leaves of *Arabidopsis thaliana*. PhD thesis. ETH Zürich, Zürich, Switzerland

[kiab468-B64] Stitt M , Zeeman SC (2012) Starch turnover: pathways, regulation and role in growth. Curr Opin Plant Biol 15: 282–292 22541711 10.1016/j.pbi.2012.03.016

[kiab468-B65] Streb S , Eicke S, Zeeman SC (2012) The simultaneous abolition of three starch hydrolases blocks transient starch breakdown in *Arabidopsis*. J Biol Chem 287: 41745–41756 23019330 10.1074/jbc.M112.395244 PMC3516724

[kiab468-B66] Thalmann M , Pazmino D, Seung D, Horrer D, Nigro A, Meier T, Kölling K, Pfeifhofer HW, Zeeman SC, Santelia D (2016) Regulation of leaf starch degradation by abscisic acid is important for osmotic stress tolerance in plants. Plant Cell 28: 1860–1878 27436713 10.1105/tpc.16.00143 PMC5006701

[kiab468-B67] Thalmann M , Coiro M, Meier T, Wicker T, Zeeman SC, Santelia D (2019) The evolution of functional complexity within the β-amylase gene family in land plants. BMC Evol Biol 19: 66 30819112 10.1186/s12862-019-1395-2 PMC6394054

[kiab468-B68] Vajravijayan S , Pletnev S, Mani N, Pletneva N, Nandhagopal N, Gunasekaran K (2018) Structural insights on starch hydrolysis by plant β-amylase and its evolutionary relationship with bacterial enzymes. Int J Biol Macromol 113: 329–337 29481953 10.1016/j.ijbiomac.2018.02.138

[kiab468-B69] Valerio C , Costa A, Marri L, Issakidis-Bourguet E, Pupillo P, Trost P, Sparla F (2011) Thioredoxin-regulated β-amylase (BAM1) triggers diurnal starch degradation in guard cells, and in mesophyll cells under osmotic stress. J Exp Bot 62: 545–555 20876336 10.1093/jxb/erq288 PMC3003804

[kiab468-B70] Vandesompele J , De Preter K, Pattyn F, Poppe B, Van Roy N, De Paepe A, Speleman F (2002) Accurate normalization of real-time quantitative RT-PCR data by geometric averaging of multiple internal control genes. Genome Biol 3: research0034.1-0034.11 12184808 10.1186/gb-2002-3-7-research0034 PMC126239

[kiab468-B71] Von Fircks Y , Sennerby-Forsse L (1998) Seasonal fluctuations of starch in root and stem tissues of coppiced *Salix viminalis* plants grown under two nitrogen regimes. Tree Physiol 18: 243–249 12651378 10.1093/treephys/18.4.243

[kiab468-B72] Wang Q , Monroe J, Sjolund RD (1995) Identification and characterization of a phloem-specific β-amylase. Plant Physiol 109: 743–750 8552713 10.1104/pp.109.3.743 PMC161373

[kiab468-B73] Wattebled F , Dong Y, Dumez S, Delvallé D, Planchot V, Berbezy P, Vyas D, Colonna P, Chatterjee M, Ball S, et al (2005) Mutants of Arabidopsis lacking a chloroplastic isoamylase accumulate phytoglycogen and an abnormal form of amylopectin. Plant Physiol 138: 184–195 15849301 10.1104/pp.105.059295 PMC1104174

[kiab468-B74] Weise SE , Kim KS, Stewart RP, Sharkey TD (2005) β-Maltose is the metabolically active anomer of maltose during transitory starch degradation. Plant Physiol 37: 756–761 10.1104/pp.104.055996 PMC1065375 15665241

[kiab468-B75] Wilkens C , Svensson B, Møller MS (2018) Functional roles of starch binding domains and surface binding sites in enzymes involved in starch biosynthesis. Front Plant Sci 9: 1652 30483298 10.3389/fpls.2018.01652 PMC6243121

[kiab468-B76] Xiao Y , Kuang J, Qi X, Ye Y, Wu ZX, Chen J, Lu W (2018) A comprehensive investigation of starch degradation process and identification of a transcriptional activator MabHLH6 during banana fruit ripening. Plant Biotechnol J 16: 151–164 28500777 10.1111/pbi.12756 PMC5785343

[kiab468-B77] Yu TS , Zeeman SC, Thorneycroft D, Fulton DC, Dunstan H, Lue WL, Hegemann B, Tung SY, Umemoto T, Chapple A, et al (2005) α-Amylase is not required for breakdown of transitory starch in Arabidopsis leaves. J Biol Chem 280: 9773–9779 15637061 10.1074/jbc.M413638200

[kiab468-B78] Zanella M , Borghi GL, Pirone C, Thalmann M, Pazmino D, Costa A, Santelia D, Trost P, Sparla F (2016) β-amylase 1 (BAM1) degrades transitory starch to sustain proline biosynthesis during drought stress. J Exp Bot 67: 1819–1826 26792489 10.1093/jxb/erv572

