# Supplementary material for: BETA-AMYLASE9 is a plastidial nonenzymatic regulator of leaf starch degradation
Source: Plant Physiol. 2021 Oct 18;188(1):191–207. doi: 10.1093/plphys/kiab468 (PMC8774843; doi:10.1093/plphys/kiab468)
Supplement: kiab468_Supplementary_Data [file kiab468_supplementary_data.zip › kiab468-suppl_data/Supplemental Figures_S1S7.pdf]

Supplemental Figures S1-S7, David et al. (2021) BETA-AMYLASE 9 regulates leaf starch degradation.

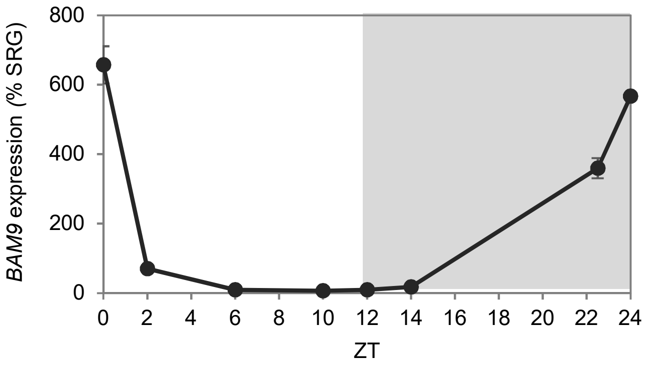

Supplemental Figure S1. *BAM9* expression over the diel cycle. Two housekeeping genes were used to normalize expression (Synthetic Relative Gene (SRG): *RHIP* and *YSL8*). N=3 biological replicates ± SE, ZT, zeitgeber time (h).

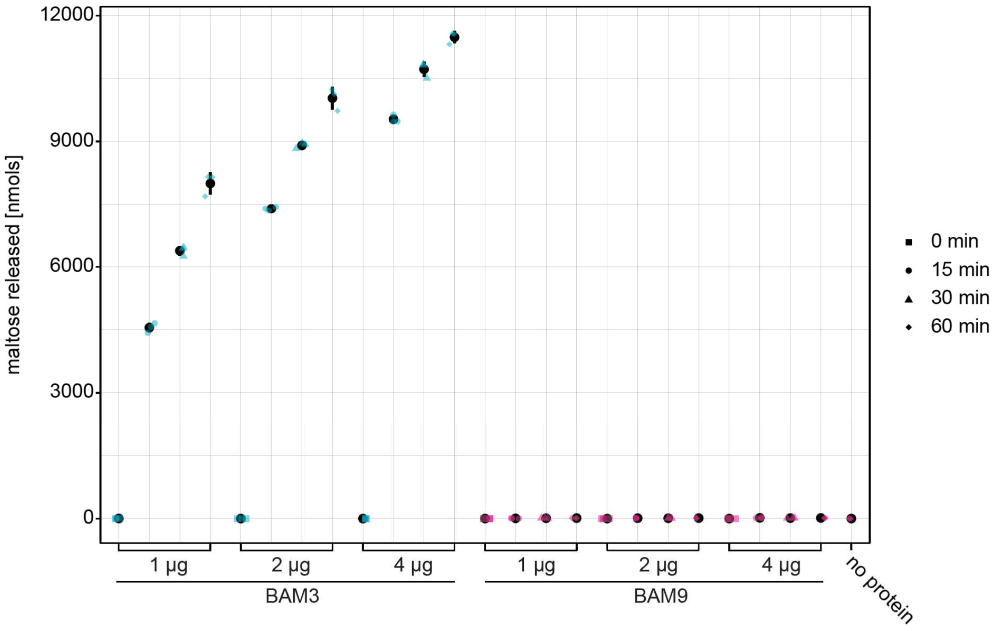

Supplemental Figure S2. Activity of recombinant BAM3 and BAM9 proteins when incubated with soluble starch. The release of maltose from soluble starch by the given quantities of recombinant proteins, incubated for the specified times was quantified by HPAEC-PAD. N = 5 experimental replicates ± SE. Note that for BAM3 the reaction saturates with prolonged incubation, particularly for the longer incubations, due to substrate depletion. Calculating a rate from the shortest incubation with the lowest BAM3 protein amount gives a specific activity (303 nmol maltose µg^{-1} protein min^{-1}) almost 1000-fold higher than against Betamyl-3 reagent presented in Figure 4 (330 pmol PNP µg^{-1} protein min^{-1}).

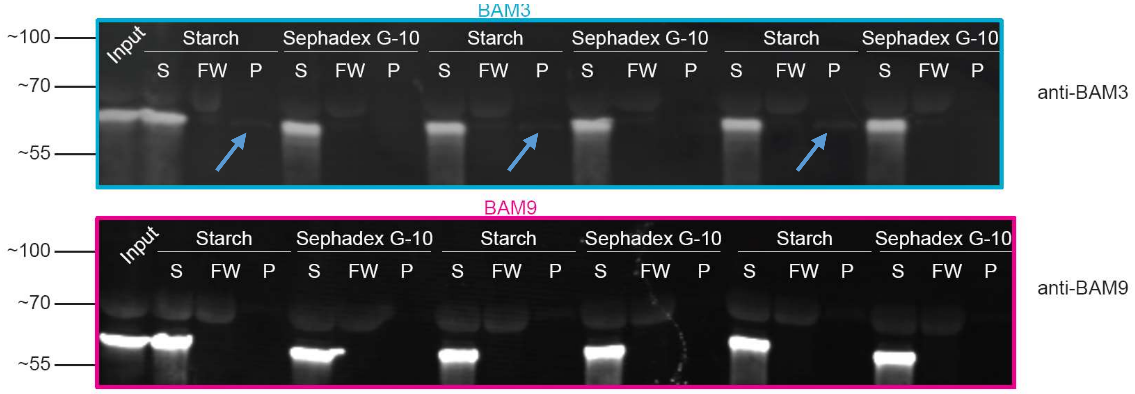

Supplemental Figure S3. Binding of recombinant BAM3 and BAM9 proteins to starch granules. Incubation of His-BAM3 and His-BAM9 recombinant proteins with intact maize starch granules *in vitro*. Binding to starch was assessed by SDS-PAGE and immunoblot analysis of soluble (S) unbound proteins, proteins in the final wash (FW) and bound proteins in the pellet (P) using anti-BAM9 antibodies (this work) or Anti-BAM3 antibodies (Fulton et al., 2008). A similar assay was performed with Sephadex G10 resin as a non-starch control substance. The cyan arrows indicate the small amounts of His-BAM3 associating with the starch. No His-BAM9 protein was detected. In repetitions of this experiment, variable results were obtained, and occasionally some BAM9 binding could sometimes be detected, albeit in small amounts. Folding of the recombinant proteins (Fig. 4B) was not confirmed in these other experiments. Nevertheless, we cannot formally rule out than BAM9 may bind with low affinity to starch.

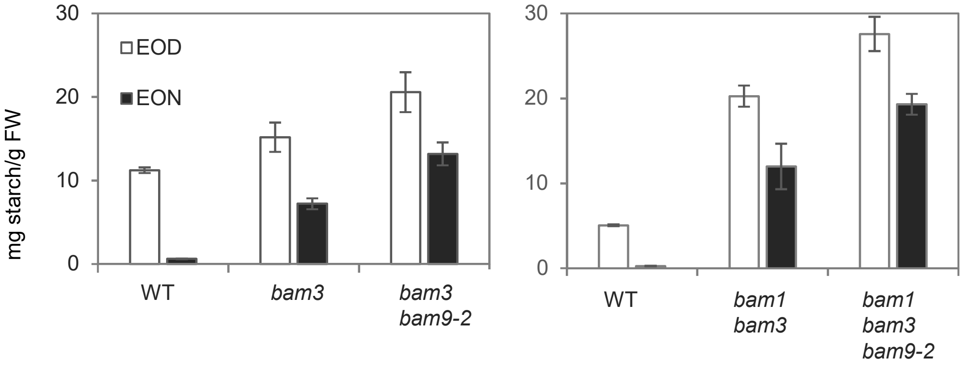

Supplemental Figure S4. Analysis of the starch contents of multiple mutants produced using the *bam9-2* mutant allele. Starch content in rosettes of 23-day-old Arabidopsis plants of the indicated genotypes, grown in 12-h day/12-h night cycle, and harvested at the end of the day (EOD) and the end of the night (EON). N = 4-5 biological replicates ± SE.

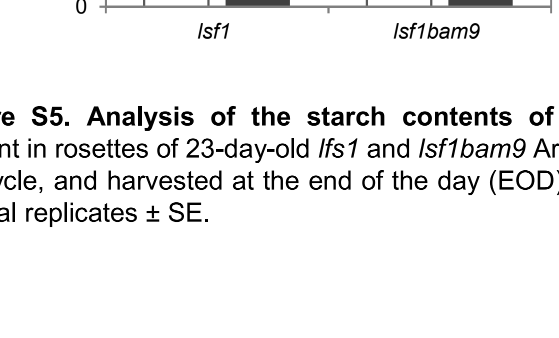

Supplemental Figure S5. Analysis of the starch contents of the *bam9 lsf1* double mutant. Starch content in rosettes of 23-day-old *lfs1* and *lsf1bam9* Arabidopsis plants grown in 12-h day/12-h nigh cycle, and harvested at the end of the day (EOD) and the end of the night (EON). N = 4 biological replicates ± SE.

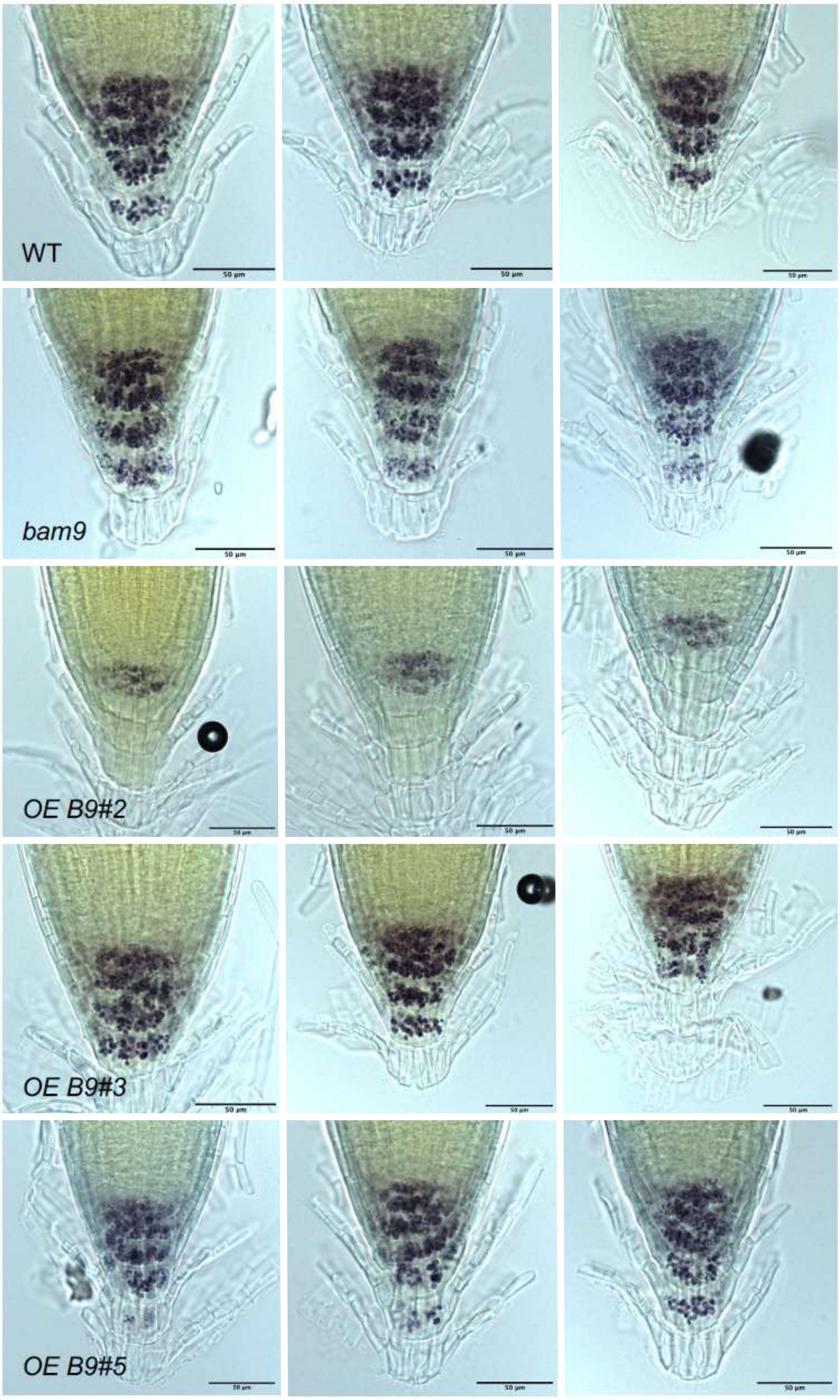

Supplemental Figure S6. Starch in the root-tip columella cells of wild type, *bam9* mutant and BAM9-overexpressing plants. The root tips of 13-day-old seedlings grown were stained with Lugol's solution, cleared and imaged by light microscopy. Three biological replicate plants were taken for each genotype pictured (bar = 50 μm).

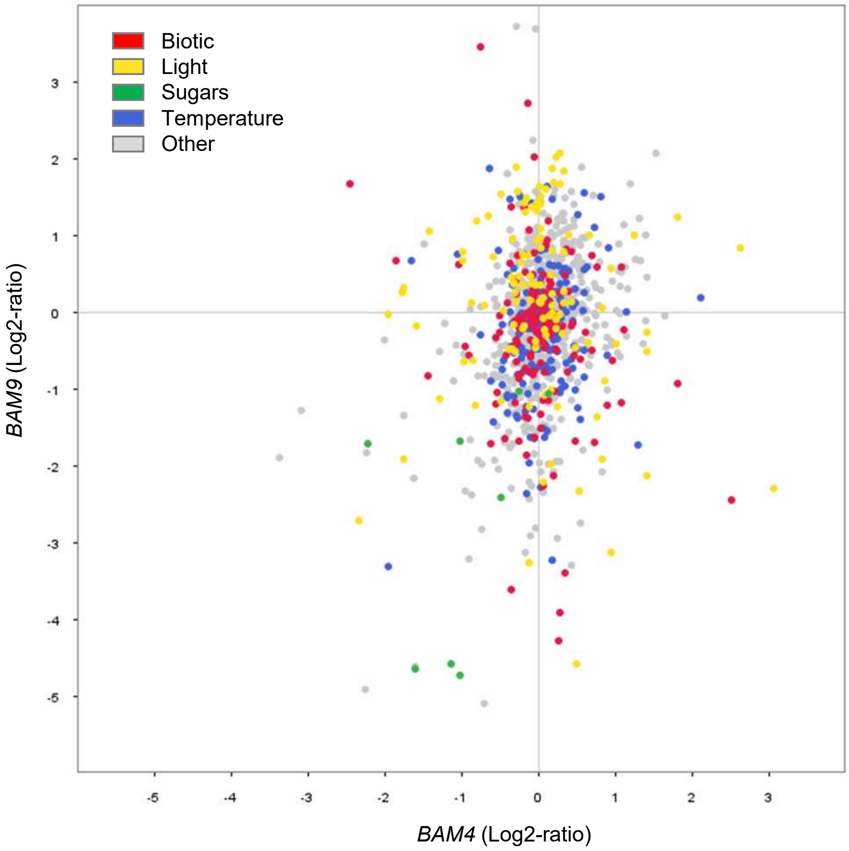

Supplemental Figure S7. Response of *BAM9* and *BAM4* gene transcription upon environmental perturbations. Data were retrieved from Genevestigator. Transcriptomic responses of wild-type plants were analysed, representing 1157 perturbations from the AT_AFFY_ATH1-1 dataset. Perturbation from similar categories have been colour-coded for more clarity. Note the higher variation in *BAM9* expression relative to *BAM4* expression.
